# Estradiol increases risk of topoisomerase IIβ-mediated DNA strand breaks to initiate Xp11.2 translocation renal cell carcinoma

**DOI:** 10.1186/s12964-021-00790-3

**Published:** 2021-11-16

**Authors:** Qiancheng Shi, Ning Liu, Lei Yang, Yi Chen, Yanwen Lu, Hongqian Guo, Xiaodong Han, Dongmei Li, Weidong Gan

**Affiliations:** 1 Department of Urology, Affiliated Drum Tower Hospital, Medical School of Nanjing University, Nanjing, Jiangsu China; 2 Department of Urology, Nanjing First Hospital, Nanjing Medical University, Nanjing, Jiangsu China; 3 Immunology and Reproduction Biology Laboratory and State Key Laboratory of Analytical Chemistry for Life Science, Medical School, Nanjing University, Nanjing, Jiangsu China; 4 Jiangsu Key Laboratory of Molecular Medicine, Nanjing University, Nanjing, Jiangsu China

**Keywords:** Renal cell carcinoma, Xp11.2, Estradiol, Estrogen receptor, Topoisomerase, TFE3

## Abstract

**Background:**

Xp11.2 translocation renal cell carcinoma (tRCC) is defined by translocation of the transcription factor E3 (*TFE3*) gene located on chromosome Xp11.2. Due to the high incidence in women, 17β-estradiol (E2) may be a factor influencing *TFE3* breaks, and the topoisomerase II (TOP2) poison is considered one of the important risk factors in mediating DNA breaks. In this study, we investigated the potential pathogenesis of Xp11.2 tRCC using the renal epithelial cell line HK-2.

**Methods:**

Immunofluorescence assay was performed to analyze DNA breaks by quantifying phosphorylation of H2AX (γH2AX), and the micronuclei (MN) assay was designed for monitoring chromosome breaks. The chromatin immunoprecipitation (CHIP) was used to detect whether proteins bound to specific DNA site, and the co-immunoprecipitation (Co-IP) was used to confirm whether proteins bound to other proteins. In some experiments, siRNA and shRNA were transfected to knockdown target genes.

**Results:**

Our results demonstrated that DNA double-strand breaks were mediated by TOP2β in HK-2 cells, and this process could be amplified through estrogen receptor α (ERα)-dependent pathway induced by E2. After performing translocation site analysis using target region sequencing data in two Xp11.2 tRCC cell lines and ten Xp11.2 tRCC patients, we confirmed that TOP2β and ERα could both bind to *TFE3* translocation sites directly to mediate DNA breaks in a E2-dependent manner. However, TOP2β and ERα were not observed to have direct interaction, indicating that their collaborative may be implemented in other ways. Besides, *TFE3* was found to be upregulated through NRF1 with increasing E2 concentration, which could increase the risk of *TFE3* breaks.

**Conclusion:**

Our results indicate that E2 amplifies TOP2β-mediated *TFE3* breaks by ERα-dependent pathway, and E2 upregulates *TFE3* by NRF1 to increase the risk of *TFE3* breaks. This suggests that E2 is an important pathogenic factor for Xp11.2 tRCC pathogenesis.

**Video Abstract**

**Supplementary Information:**

The online version contains supplementary material available at 10.1186/s12964-021-00790-3.

## Background

Xp11.2 translocation renal cell carcinoma (tRCC), first reported in 1988, is characterized by a pathognomonic chromosomal translocation of transcription factor E3 (*TFE3*), which causes fusion of the *TFE3* gene with a variety of partner genes [1, 2]. Children and young adults are most often affected, for 46.7% of paediatric RCCs and 15% of young adult (aged < 45 years) RCCs are Xp11.2 tRCC, and the peak age of onset is 20–29 years [3–5]. Due to its aggressiveness, Xp11.2 tRCC is characterized by poor prognosis with local invasive and distant metastases [6–9], and the high prevalence of young adults increased the medical care related to complications of this disease in recent years. Another important feature of Xp11.2 tRCC is the female predominance. The female/male ratio of Xp11.2 tRCC is 1.68–2 [10, 11], which is totally different from common RCCs [12]. Considering age- and sex-specific factors of this type of RCC, we suspect that estrogen may play an important role in the pathogenesis of Xp11.2 tRCC.

The topoisomerase II (TOP2) poison has been reported to be a causal agent of this type of RCC, for patients could have a history of TOP2 poisons (etoposide or doxorubicin) use before diagnosed with Xp11.2 tRCC [13–15]. These drugs are used in the treatment of a variety of cancers as a broad class of chemotherapeutic agents [16]. The role of TOP2-mediated DNA double-strand breaks in translocation tumors especially MLL leukemia has been widely reported [17, 18]. However, whether the specific *TFE3* gene breaks in Xp11.2 tRCC are produced via TOP2-mediated DNA double-strand breaks is still unclear. Exploring the TOP2-mediated DNA double-strand breaks at *TFE3* translocation sites has important implications in understanding the pathogenesis of Xp11.2 tRCC.

Since 17β-estradiol (E2) and estrogen receptor (ER)-mediated DNA breaks have been reported [19, 20], we consider that E2 and ER may induce *TFE3* breaks through TOP2-mediated DNA double-strand breaks in Xp11.2 tRCC. Thus, this study was designed to confirm the role of TOP2-mediated DNA double-strand breaks in *TFE3* translocation sites of Xp11.2 tRCC, and the regulatory functions of E2 and ER in this process.

## Materials and methods

### Cell culture

HK-2 cells were obtained from ATCC (ATCC^®^ CRL-2190™), and then authenticated by STR profiling at Shanghai Zhong Qiao Xin Zhou Biotechnology Co., Ltd. These HK-2 cells were cultured in base medium provided by Invitrogen (GIBCO) supplemented with 0.05 mg/ml bovine pituitary extract (BPE) and 5 ng/ml human recombinant epidermal growth factor (EGF) in 5% CO_2_ at 37 °C. For etoposide stimulation, HK-2 cells were plated in base medium with 0.05 mg/ml BPE and 5 ng/ml EGF for a minimum of 16 h before being washed 3 times in 1 × PBS. The same medium was then added for 1 h prior to the addition of etoposide dissolved in DMSO or the vehicle control (100% DMSO). For estrogen stimulation, HK-2 cells were pre-processed using the same approach as mentioned earlier, and the same medium with E2 dissolved in DMSO or the vehicle control were then added for 24 h or 48 h. UOK109 and UOK120 cells were gifts of Dr. W. Marston Linehan, National Cancer Institute, Bethesda, MD. The UOK109 and UOK120 cell lines were derived from primary RCC as described [21], and were derived from tumors arising in a 30- and a 39-year-old male, respectively. These UOK109 and UOK120 cells were cultured in DMEM (GIBCO) supplemented with 10% FBS in 5% CO_2_ at 37 °C.

### Antibodies, plasmids, reagents, and primers

Antibodies to P-H2AX (Cell Signaling, 80312S), TOP2β (Abcam, ab72334), CTCF (Abcam, ab128873), ER-α (Cell Signaling, 8644S), and Alexa Fluor Plus 488 (Invitrogen, A32723) were used. Two siRNA were used to deplete *TOP2A* and *TOP2B* separately, and NC siRNA was used for negative control. Three shRNA were used to knockdown *ESR1*, *ESR2* and *NRF1* separately, and NC shRNA was used for negative control. The siRNA sequences and shRNA sequences can be found in the supplementary materials [see Additional file 1]. All siRNA transfections were performed using Lipofectamine 2000™ (Thermo Fisher, 11668019) according to the manufacturer’s protocol and 20 nM siRNA. All lentiviral vectors expressing the scramble shRNA transfections were performed using polybrene (Sigma-Aldrich, TR-1003). Etoposide (Sigma-Aldrich, E1383) and E2 (Sigma-Aldrich, E8875) was dissolved in DMSO (Sigma-Aldrich, D2650). All primers and primer sequences can be found in the supplementary materials [see Additional file 1].

### Relative quantitative real-time polymerase chain reaction

The action mixture consisted of 10 μl SYBR Green (Vazyme, Nanjing, Q711-02), 10 μM each primer, 1 μl cDNA. PCR amplifications were performed on the 7300 real-time PCR system (Applied Biosystems, CA, US). The relative mRNA expression level was calculated by the comparative 2^−ΔΔCt^ method and normalized against ACTB mRNA. For reverse transcription-PCR analysis, amplification was done for 33 cycles, each with denaturation at 95 °C for 30 s, annealing at 60 °C for 30 s and extension at 72 °C for 30 s.

### Micronuclei assays

Cytochalasin B (MCE, HY-16928) and Acridine Orange (Sigma-Aldrich, A6014) were used for micronuclei assays as reported in literature [22].

### Fluorescence in-situ hybridization (FISH)

FISH was performed using FISH Tag DNA Green Kit (Thermo Fisher, F32947).

BAC clones CTD-2516D6 (*TFE3*-5′), CTD-2522M13 (*TFE3*-5′), RP11-416814 (*TFE3*-5′), CTD-2312C1 (*TFE3*-3′), CTD-2248C21 (*TFE3*-3′), RP11-959H17 (*TFE3*-3′) were selected from The UCSC Genome Browser (http://genome.ucsc.edu). Probes were labeled with FITC (all 5′ probes) and TexasRed (all 3′ probes) conjugated dUTP (C7604, C7631, Invitrogen). The fusion signal appears as one yellow signal superimposed by one red and one green signal together, and the separated signal appears as the distance between the red and the green signals more than one signal point diameter. Since the female cell line we used contain two X chromosomes, the negative result presents two yellow fusion signals, and the positive result presents one yellow fusion signal and two separate signals (one red and one green).

### Chromatin immunoprecipitation (ChIP)

Chromatin immunoprecipitation was performed using Pierce Magnetic ChIP Kit (Thermo Fisher, 26157). The HK-2 cells were crosslinked using formaldehyde with a final concentration of 1%, and the number of cells required 2 × 10^6^.

### Co-immunoprecipitation (Co-IP)

Co-IP was performed using Pierce Co-Immunoprecipitation Kit (Thermo Fisher, 26149). For each IP, 8 μg antibody (ER-α (Cell Signaling, 8644S)) was added.

## Results

### E2 induces TFE3 breaks in HK-2 cells

**Fig. 1.**
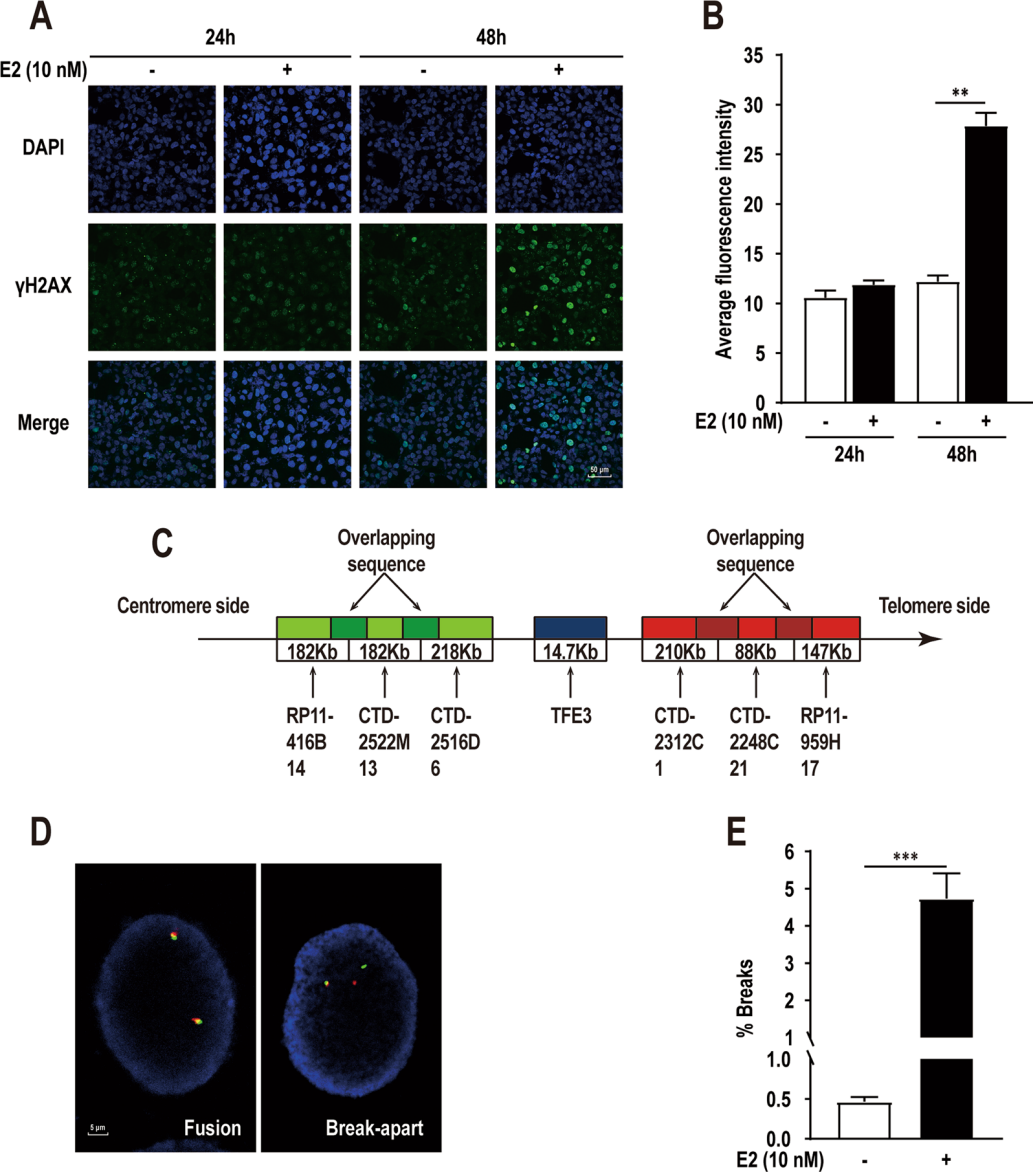
17β-estradiol (E2)-induced DNA breaks and *TFE3* breaks analysis. **A** Phosphorylation of H2AX (γH2AX) focis detected in HK-2 cells under 0.1% DMSO (solvent-only control) or 10 nM E2 treatment for 24 h or 48 h. **B** Quantification of the γH2AX focis in E2-treated cells as in panel **A**. **C** Pattern diagram of *TFE3* break-apart rearrangement probe. **D** Examples of fusion and break-apart signal using *TFE3* break-apart breaksfluorescence in situ hybridization (FISH) probe. **E** Quantification of the *TFE3* break-apart signals in HK-2 cells under 0.1% DMSO (solvent-only control) or 10 nM E2 treatment for 48 h. Error bars indicate 95% confidence intervals (**p < 0.01; ***p < 0.001)

Considering the potential oncogenic role of E2 in the pathogenesis of Xp11.2 tRCC, we assessed the ability of E2 to induce DNA breaks. The renal epithelial cell line HK-2 was treated with E2 at a concentration of 10 nM, and the histone H2AX phosphorylation was used as an indicator for reflecting DNA double-strand breaks. When using immunofluorescent staining to detect phosphorylated H2AX (γ-H2AX) foci, E2 induced remarkably breaks more than control group after 48 h (Fig. 1A, B). To directly assess the role of E2 in generating breaks at the *TFE3* locus, we used DNA-FISH with a “break-apart” rearrangement probe to quantify *TFE3* breaks (Fig. 1C, D). E2 could induce *TFE3* breaks significantly after 48 h at the concentration of 10 nM in HK-2 cells (Fig. 1E). The results confirm the role of E2 in *TFE3* breaks.

### TOP2β mediates DNA double-strand breaks

**Fig. 2:**
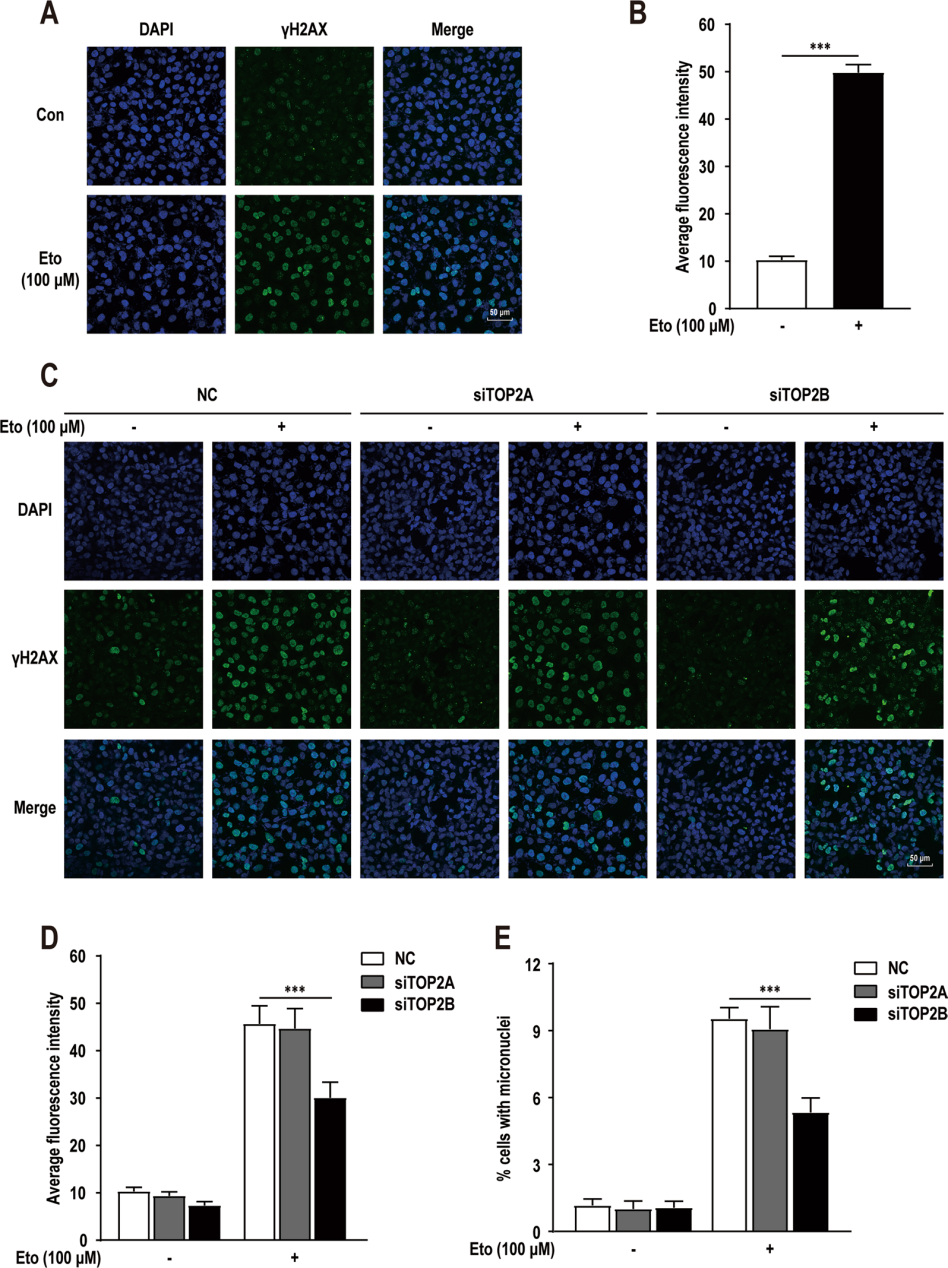
Etoposide-induced topoisomerase II (TOP2)-mediated DNA breaks and chromosomal breaks. **A** γH2AX focis detected in HK-2 cells under 0.1% DMSO (solvent-only control) or 100 μM etoposide treatment for 1 h. **B** Quantification of the γH2AX focis in etoposide-treated cells as in panel **A**. **C** γH2AX focis detected in HK-2 cells transfected with negative control siRNA (NC), siRNA against *TOP2A* (siTOP2A), or siRNA against *TOP2B* (siTOP2B) under 0.1% DMSO (solvent-only control) or 100 μM etoposide treatment for 1 h. **D** Quantification of the γH2AX focis in etoposide-treated cells as in panel **C**. **E** Quantification of the micronucleis (MNs) in etoposide-treated cells as in panel **C**. Error bars indicate 95% confidence intervals (***p < 0.001)

To ascertain the role of TOP2-mediated DNA cleavage in renal carcinogenesis with chromosomal translocation, HK-2 cells were treated with the TOP2 poison etoposide at a concentration of 100 μM for a short time period (1 h) (Additional file 4: Fig. S1A). Results showed that etoposide induced remarkably breaks more than control group (Fig. 2A, B). To determine which of the two TOP2 affected the etoposide-induced DNA damage across the genome, HK-2 cells were transfected with negative control siRNA (NC), siRNA against *TOP2A* (siTOP2A), or siRNA against *TOP2B* (siTOP2B) (Additional file 4: Fig. S1C). The immunofluorescence analysis showed similar results, DNA breaks of each group were extensively increased after etoposide treatment, but only *TOP2B* knockdown significantly decreased the γ-H2AX foci (Fig. 2C, D), indicating that *TOP2B* knockdown decreased DNA breaks. To verify the contribution made by TOP2 to etoposide-induced genotoxicity at the chromatin level, we next carried out micronuclei (MN) assays (Additional file 4: Fig. S1D). The *TOP2A* knockdown group behaved in the same way as control group, whereas in *TOP2B* knockdown group there was a significant decrease in the MN frequency of etoposide-treated cells (Fig. 2E). These results show that TOP2β mediates DNA double-strand breaks in HK-2 cells, supporting the role for TOP2β in the genotoxic effects of etoposide.

### TOP2β mediates TFE3 breaks directly

**Fig. 3:**
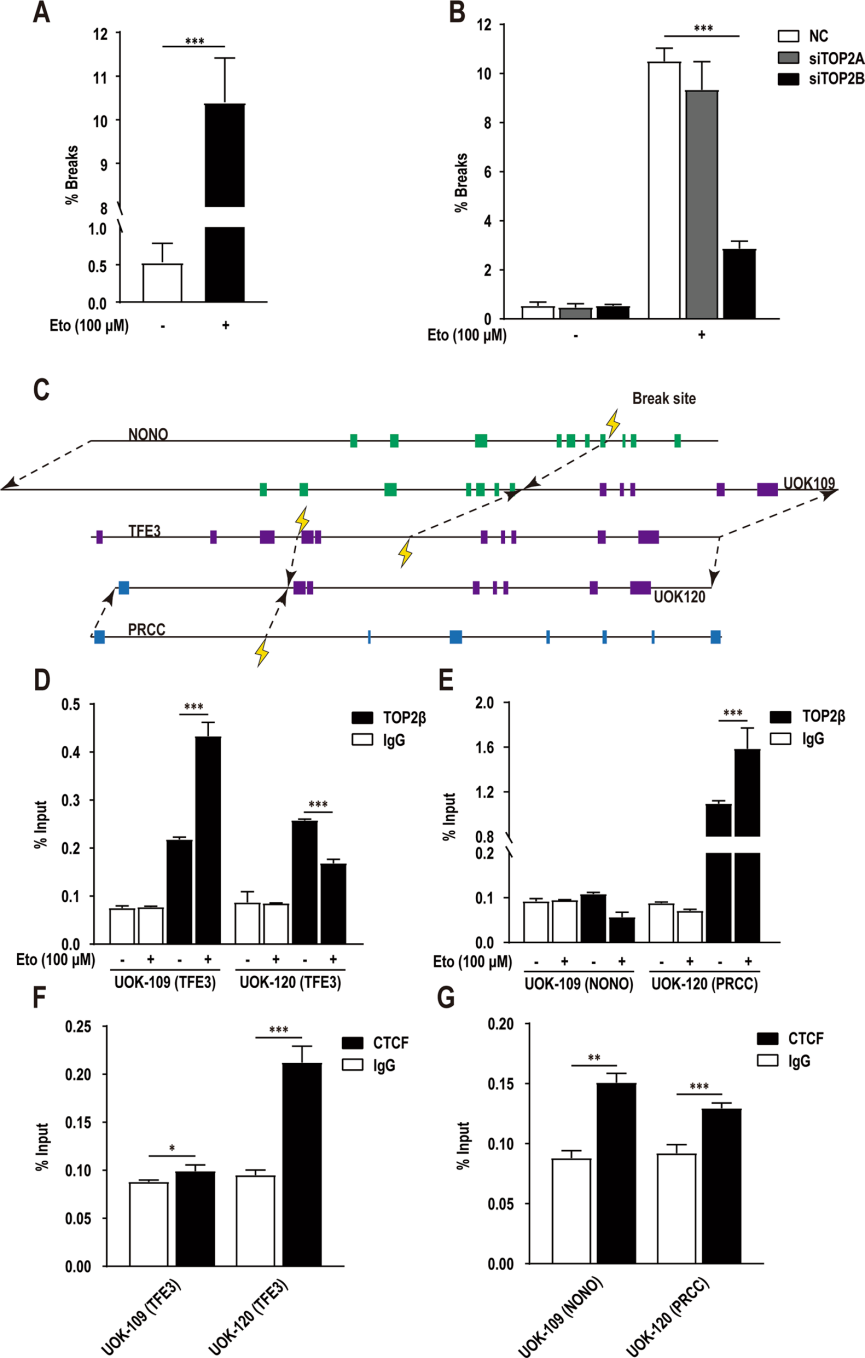
Etoposide-induced TOP2-mediated *TFE3* breaks in Xp11.2 translocation renal cell carcinoma (tRCC) translocation sites. **A** Quantification of the *TFE3* break-apart signals in HK-2 cells under 0.1% DMSO (solvent-only control) or 100 μM etoposide treatment for 1 h. **B** Quantification of the *TFE3* break-apart signals in HK-2 cells transfected with NC or siTOP2B under 0.1% DMSO (solvent-only control) or 100 μM etoposide treatment for 1 h. **C** Ideograms of the translocation fragments of two Xp11.2 tRCC cell lines, UOK109 and UOK120. **D** and **E** Statistics of TOP2β chromatin immunoprecipitation (ChIP)-qPCR results performed using the primers near the translocation sites as in panel **c** in HK-2 cells under 0.1% DMSO (solvent-only control) or 100 μM etoposide treatment for 1 h. **F** and **G** Statistics of CTCF ChIP-qPCR results performed using the same primers as in panel **D** and **E** in HK-2 cells. ChIP-qPCR was normalized to Input DNA, experiments were repeated three times and data shown are means ± SD. Error bars indicate 95% confidence intervals (*p < 0.05; **p < 0.01; ***p < 0.001)

To further clarify the specific mechanism of *TFE3* breaks in Xp11.2 tRCC, we used DNA-FISH with a “break-apart” rearrangement probe as above described. Results showed that etoposide induced approximately 20-fold breaks at the *TFE3* locus in HK-2 cells, and breaks in the same position was substantially reduced in *TOP2B* knockdown group (Fig. 3A, B). Subsequently, DNA sequences of the translocation site were analyzed via target region sequencing in two Xp11.2 tRCC cell lines, UOK109 and UOK120 (Fig. 3C and Additional file 1). We used ChIP-qPCR to determine the distribution of TOP2β over the translocation sites in UOK109 and UOK120 cells, respectively. The results of sampling multiple translocation sites in Xp11.2 tRCC revealed the presence of TOP2β in chromatin in most of the positions sampled (Fig. 3D, E). The same sites were analyzed in cells treated with 100 μM etoposide for 60 min, which resulted in a large increase in stabilized covalent TOP2β DNA complexes. Because CTCF has been reported to bind at anchors of chromatin loops, where requires TOP2 for the initiation of transcription [23], we measured CTCF binding in the same way (Fig. 3F, G). Sampling CTCF binding in the same positions confirmed the presence of a CTCF binding. This is further proof that the *TFE3* breaks in Xp11.2 tRCC are a result of TOP2β-mediated DNA breaks.

### E2 induces TFE3 breaks through ERα-dependent pathway

**Fig. 4:**
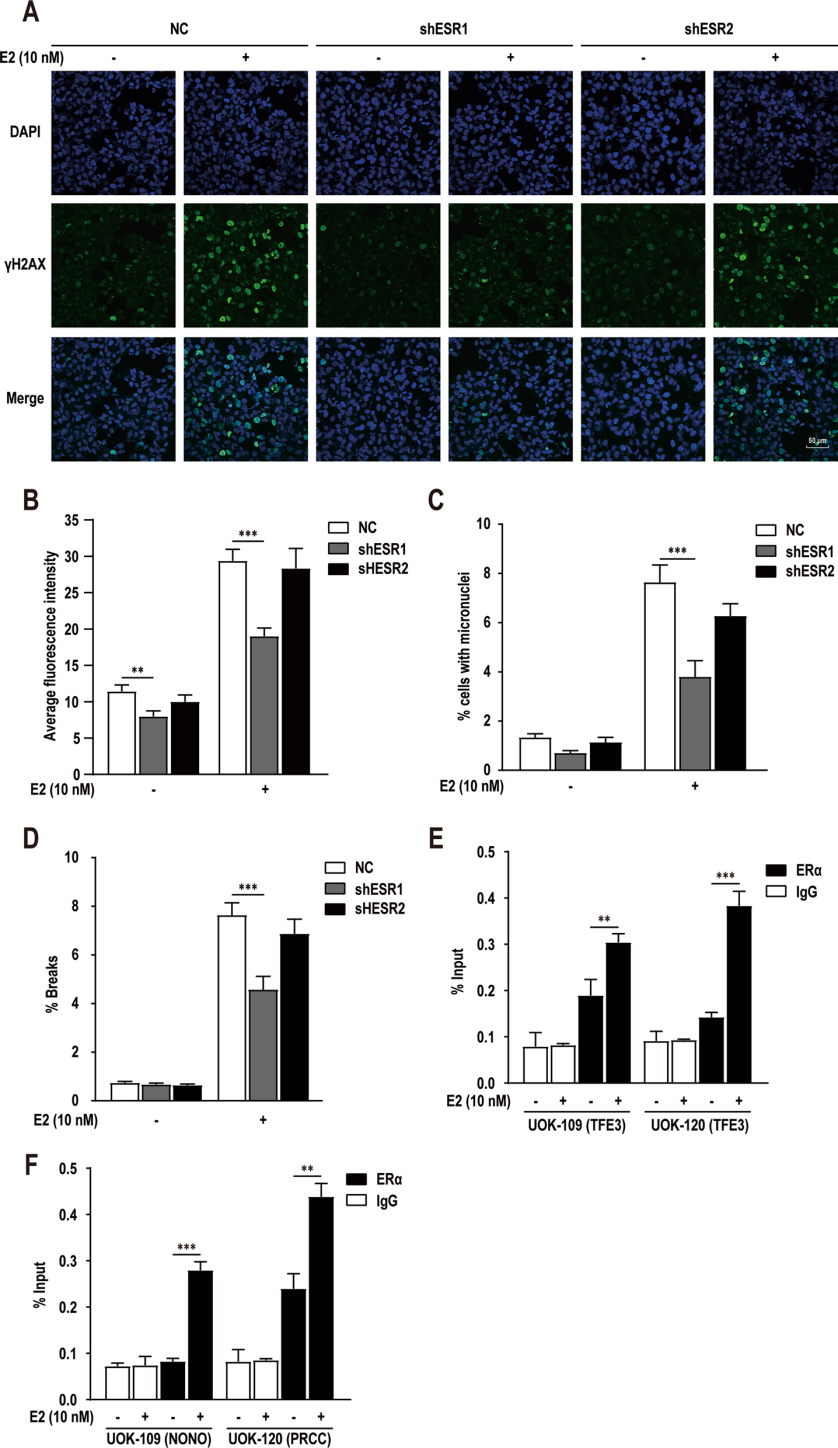
E2 induced-*TFE3* breaks through ERα-dependent pathway. **A** γH2AX focis detected in HK-2 cells transfected with negative control shRNA (NC), shRNA against *ESR1* (shESR1), or shRNA against *ESR2* (shESR2) under 0.1% DMSO (solvent-only control) or 10 nM E2 treatment for 48 h. **B** Quantification of the γH2AX focis in E2-treated cells as in panel **A**. **C** Quantification of the MNs in E2-treated cells as in panel **A**. **D** Quantification of the *TFE3* break-apart signals in HK-2 cells transfected with NC or shESR1 under 0.1% DMSO (solvent-only control) or 10 nM E2 treatment for 48 h. **E** and **F** Statistics of TOP2β ChIP-qPCR results performed using the primers near the translocation sites in HK-2 cells under 0.1% DMSO (solvent-only control) or 10 nM E2 treatment for 48 h. ChIP-qPCR was normalized to Input DNA, experiments were repeated three times and data shown are means ± SD. Error bars indicate 95% confidence intervals (**p < 0.01; ***p < 0.001)

To assess whether the effects of E2 are mediated via ER, we constructed shRNA-lentivirus to knockdown *ESR1* or *ESR2* expression in HK-2 cells (Additional file 4: Fig. S1G), and repeated the above described experiment using *ESR1* and *ESR2* knockdown cells. Quantification of γ-H2AX showed that the increase of DNA breaks induced by E2 was disappeared after knockdown of *ESR1* (Fig. 4A, B), Similar results were also observed in subsequent MN assays (Fig. 4C). In order to investigate whether ERα were associated with *TFE3* breaks in Xp11.2 tRCC, we used DNA-FISH with a “break-apart” rearrangement probe as above described. Results revealed that E2-induced *TFE3* breaks were markedly attenuated in *ESR1* knockdown cells (Fig. 4D). The results show that E2 induces *TFE3* breaks through ERα-dependent pathway.

To verify this, ChIP-qPCR experiments were performed in HK-2 cells with the specific anti-ERα antibodies validated by ChIP-qPCR. The results showed that ERα bound to multiple sites near the translocation site of *TFE3* and other partner genes (*NONO* and *PRCC*) (Fig. 4E, F). After induction with E2 (10 nM), a substantial increase in binding intensities of ERα was observed at these sites. These results reveal that ERα induce *TFE3* breaks directly.

### TOP2β and ERα have a combined effect in mediating DNA breaks

**Fig. 5:**
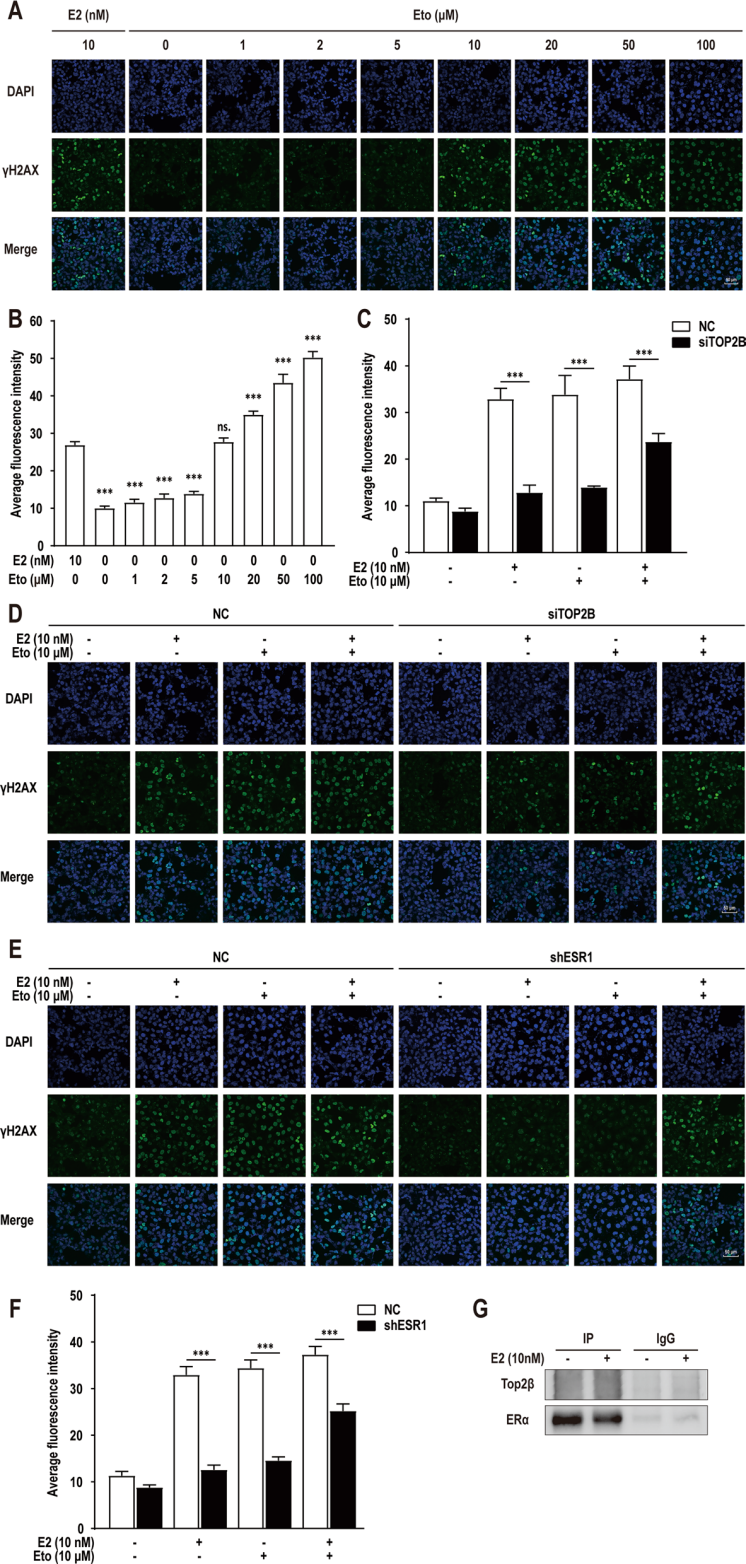
Combined effect of TOP2β and ERα in mediating DNA breaks. **A** γH2AX focis detected in HK-2 cells under 10 nM E2 treatment for 48 h or a concentration gradient of etoposide (0, 1, 2, 5, 10, 20, 50, 100 μM) for 1 h. **B** Quantification of the γH2AX focis in cells as in panel **A**. **C** and **D** γH2AX focis detected in HK-2 cells transfected with negative control siRNA (NC), siRNA against *TOP2A* (siTOP2A), or siRNA against *TOP2B* (siTOP2B) under 0.1% DMSO (solvent-only control), 10 nM E2, 10 μM etoposide or a combination of the two drugs and their quantification. **E** and **F** γH2AX focis detected in HK-2 cells transfected with negative control shRNA (NC), shRNA against *ESR1* (shESR1), or shRNA against *ESR2* (shESR2) under 0.1% DMSO (solvent-only control), 10 nM E2, 10 μM etoposide or a combination of the two drugs and their quantification. **G** Western blot of ERα co-immunoprecipitation (Co-IP) detected with anti-Top2β antibodies. Error bars indicate 95% confidence intervals (***p < 0.001)

To determine the association between E2-induced DNA breaks and TOP2β-mediated DNA breaks, HK-2 cells were treated with a concentration gradient of etoposide (0, 1, 2, 5, 10, 20, 50, and 100 μM) to determine the appropriate concentration which could induce similar numbers of breaks to 10 nM E2 treatment (Fig. 5A, B). The results showed that etoposide treatment of 0–5 μM could not induce a significant DNA breaks in HK-2 cells, and DNA breaks were significantly increased from the concentration of 10 μM. At the same time, the 10 μM etoposide treatment group showed similar DNA breaks to that of the 10 nM E2 group (*p* > 0.05). So, the concentration of 10 μM etoposide was used in the follow-up studies to explore the combined effects of these two drugs.

The *TOP2B* knockdown cells and control cells were treated with 0.1% DMSO (solvent-only control), 10 nM E2, 10 μM etoposide or a combination of the two drugs, respectively. Quantification of γ-H2AX showed that DNA breaks were significantly reduced in *TOP2B* knockdown cells whether treated with E2, etoposide or a combination of the two drugs (Fig. 5C, D). Similarly, we repeated the similar experiment using *ESR1* knockdown HK-2 cells. Results showed that DNA breaks were also significantly reduced in *ESR1* knockdown cells whether treated with E2, etoposide or a combination of the two drugs (Fig. 5E, F). Taken together, these results indicating that TOP2β and ERα play a synergistic role in the process of DNA breaks induced by E2 or etoposide.

Based on this finding, we speculated that E2 might function by modulating the interaction between ERα and TOP2β. We then performed Co-IP experiments, proteins directly bound to ERα were detected by western blot with anti-TOP2β antibodies. Unfortunately, no binding signal of TOP2β was observed in HK-2 cells with or without E2 treatment (Fig. 5G). Another explanation may be that the direct binding of ERα to *TFE3* sequence affects the structural, and possibly the functional, properties of TOP2β, resulting in the site-specific break in the translocation site of *TFE3*.

### Further validation in ten Xp11.2 tRCC patients

**Fig. 6:**
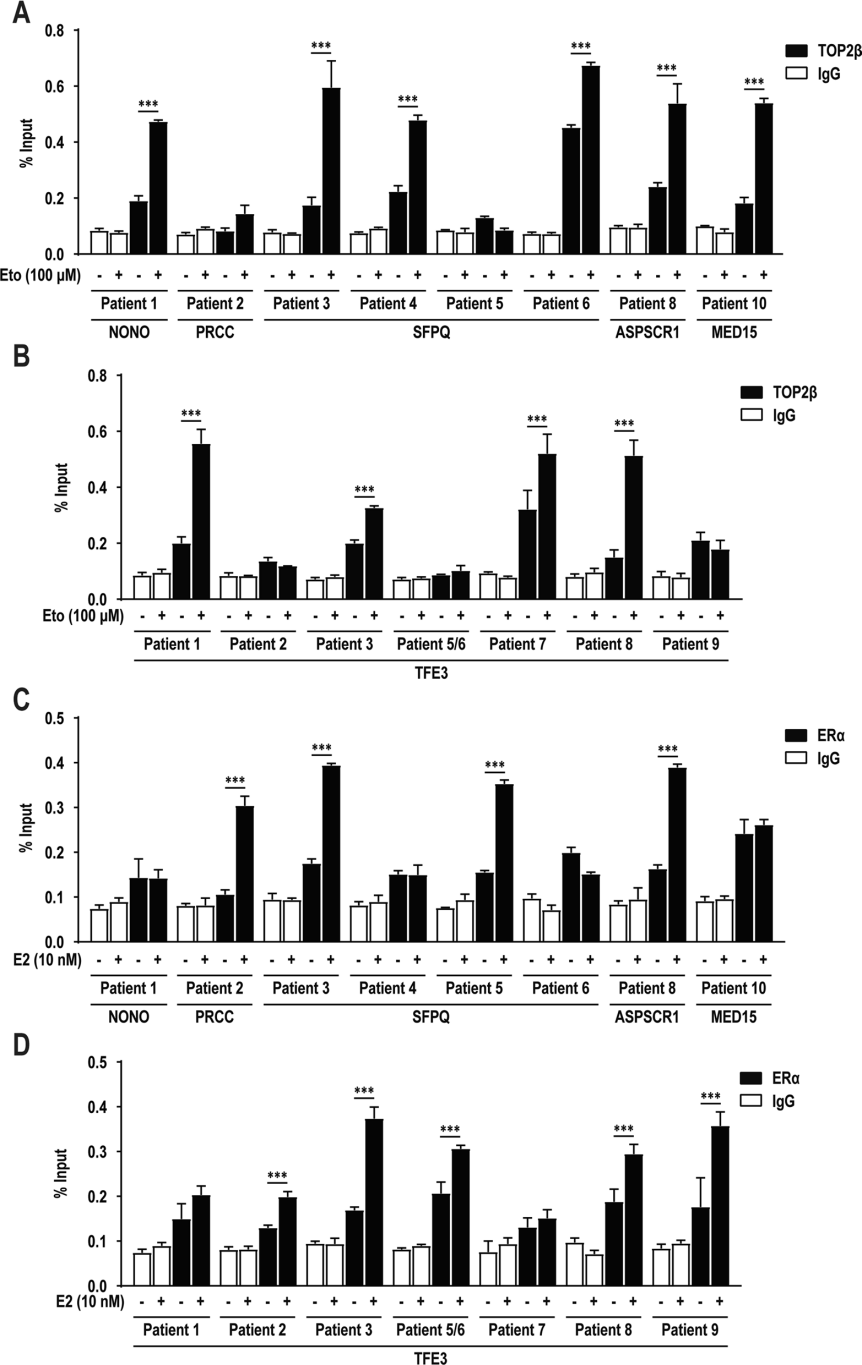
E2-induced ERα and TOP2β-dependent *TFE3* breaks in translocation sites of ten Xp11.2 tRCC patients. **A** Statistics of TOP2β ChIP-qPCR results performed using primers designed for partner genes (*NONO*, *PRCC*, *SFPQ*, *ASPSCR1* and *MED15*) in ten Xp11.2 tRCC patients in HK-2 cells under 0.1% DMSO (solvent-only control) or 100 μM etoposide treatment for 1 h. **B** Statistics of TOP2β ChIP-qPCR results performed using primers designed for *TFE3* in ten Xp11.2 tRCC patients in HK-2 cells under 0.1% DMSO (solvent-only control) or 100 μM etoposide treatment for 1 h. **C** Statistics of ERα ChIP-qPCR results performed using the same primers as in panel **A** in HK-2 cells under 0.1% DMSO (solvent-only control) or 10 nM E2 treatment for 48 h. **D** Statistics of ERα ChIP-qPCR results performed using the same primers as in panel **B** in HK-2 cells under 0.1% DMSO (solvent-only control) or 10 nM E2 treatment for 48 h. ChIP-qPCR was normalized to Input DNA, experiments were repeated three times and data shown are means ± SD. Error bars indicate 95% confidence intervals (***p < 0.001)

To further verify the conclusions drawn above, we used target region sequencing to analyze the DNA sequences of the translocation site in ten Xp11.2 tRCC patients [see Additional file 1]. And primers were designed within 100 bp before or after the translocation site, if possible. Due to the particularity of the sequence near the break site in some patients, no valuable primers can be designed for PCR operation. These specific sites were excluded from our study. In subsequent research, we consider using a more systematic high-throughput method ChIP-sequence to focus on these specific individuals. We then used ChIP-qPCR to determine the distribution of TOP2β and ERα over the translocation sites in ten Xp11.2 tRCC patients, using HK-2 cells with or without etoposide or E2 treatment respectively. The results showed that TOP2β and ERα extensively bound to multiple sites near the translocation site of *TFE3* and other partner genes (*NONO*, *PRCC*, *SFPQ*, *ASPSCR1* and *MED15*) (Fig. 6A–D). This is further proof that the *TFE3* breaks in Xp11.2 tRCC are a result of ERα and TOP2β-mediated DNA breaks.

### E2 increases the risk of TFE3 breaks through NRF1 pathway

**Fig. 7:**
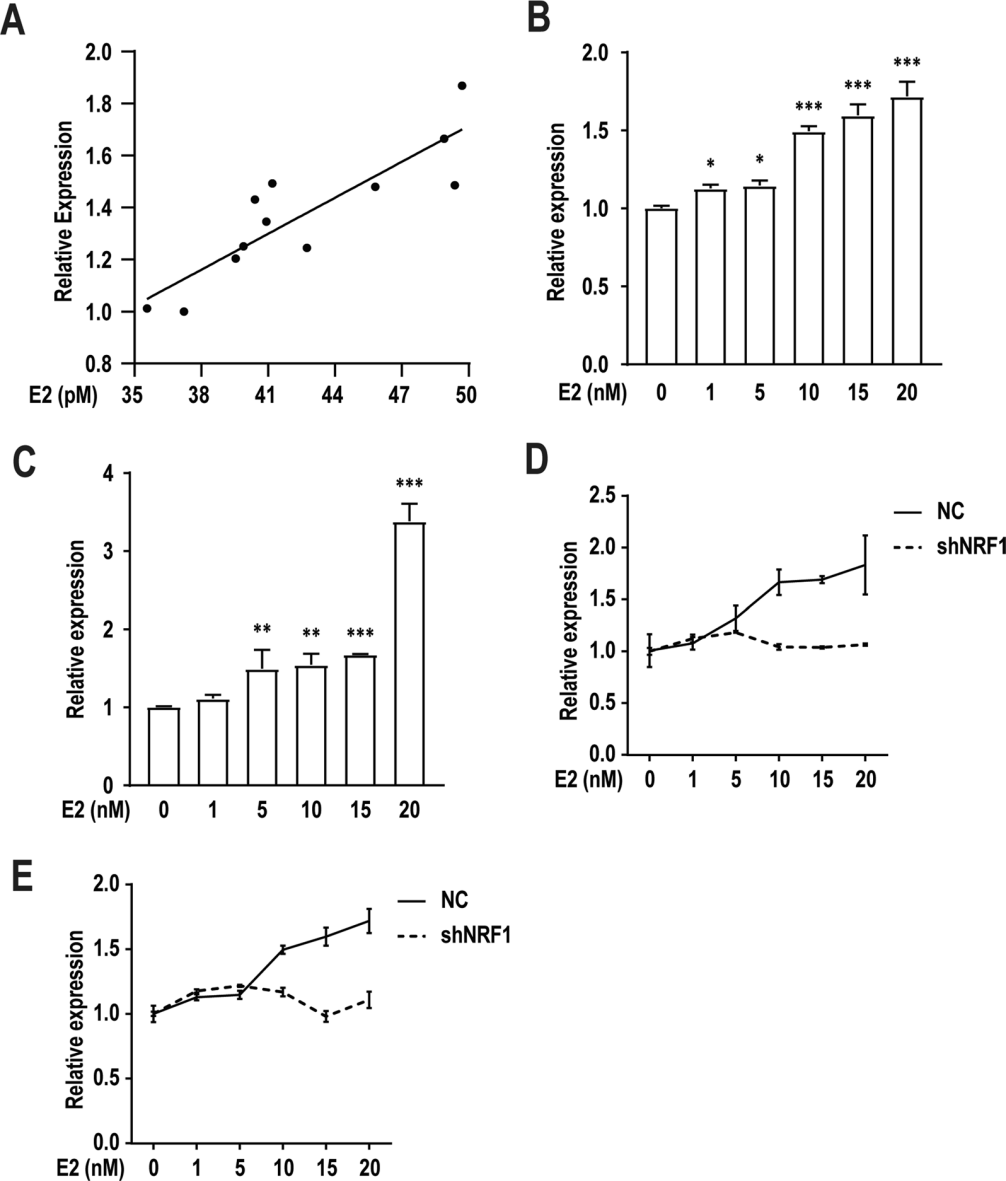
E2-induced NRF1-dependent *TFE3* expressions. **A** Correlation analysis of *TFE3* mRNA level and serum E2 concentration in BalbC mice aged 4–10 weeks. **B** Statistics of *TFE3* qPCR results in HK-2 cells under 0.1% DMSO (solvent-only control), 1 nM, 5 nM, 10 nM, 15 nM or 20 nM E2 treatment for 48 h. **C** Statistics of *ESR1* qPCR results in E2-treated cells as in panel **B**. **D** Statistics of *TFE3* qPCR results in HK-2 cells transfected with negative control shRNA (NC) or shRNA against *NRF1* (shNRF1) under the same treatment as in panel **B**. **E** Statistics of *NRF1* qPCR results in E2-treated cells as in panel **D**. Error bars indicate 95% confidence intervals (*p < 0.05; **p < 0.01; ***p < 0.001)

To investigate whether E2 can be regulated *TFE3* expression, we tested renal *TFE3* mRNA level and serum E2 concentration in BalbC mice aged 4–10 weeks. Results showed that *TFE3* expression was significantly associated with E2 concentration (Fig. 7A). We further observed that dose-dependent significant increases in *TFE3* and *ESR1* with increasing E2 concentration in HK-2 cells (Fig. 7B, C). We considered whether activation of *TFE3* expression by ERα-dependent pathway could increase the risk of *TFE3* breaks. However, there was no ERα-binding site in the promoter region of *TFE3*, predicted by using the online website *JASPAR* (http://jaspar.genereg.net/). To identify putative transcription factors that can bind to the promoter region of *TFE3*, we systematically searched databases of known human transcription factors [see Additional file 2]. Results are sorted by best relative score. Apart from a broad-spectrum binding protein to zinc finger domain, ZNF263, the highest score was given to NRF1. Since the ability of E2 to regulate *NRF1* expression has been widely confirmed by previous researches [24], we then examined whether NRF1 was involved in *TFE3* expression induced by E2. We found that *TFE3* expression was drastically reduced following *NRF1* knockdown, and the dose-dependent increases was also disappeared with increasing E2 concentration (Fig. 7D, E and Additional file 4: Fig. S1H). Together, the results show that E2 upregulates *TFE3* expression by enhancing its transcriptional activity through NRF1 pathway and consequently increases the risk of *TFE3* breaks.

## Discussion

Since Xp11.2 tRCC has been formally described as a distinct clinicopathologic entity in 2001, there has been a lot of research attention given to the function of TFE3 fusion proteins [25–27]. However, few studies have investigated the pathogenesis of Xp11.2 tRCC. Our study proposed that physiological concentrations of E2 could induce *TFE3* breaks in Xp11.2 tRCC, which explained the high morbidity in women. In this study, we confirmed that *TFE3* breaks were mediated by TOP2β, which process could be amplified through ERα-dependent pathway induced by E2. Although TOP2β and ERα could both bind to *TFE3* translocation sites directly to mediate DNA breaks, no direct interaction was observed, indicating that their collaborative may be implemented in other ways. Besides, *TFE3* was found to be upregulated through NRF1 with increasing E2 concentration, which could increase the risk of *TFE3* breaks.

Different from etoposide with rapid onset, E2 require an extended period (48 h) of treatment to induce *TFE3* breaks. On the one hand, this may because the indirectly affected of ERα-mediated DNA breaks is less prominently than acting directly on TOP2β. On the other hand, E2 induced increase in transcriptional activity of *TFE3* requires the expression of *NRF1*, resulting in a delayed *TFE3* breaks. This may explain the reason why the peak age of onset is 20–29 years instead of the early pubertal stages suffering initial exposure with high concentrations of E2 [5].

Several studies reported about the function of sex hormone in TOP2β-mediated DNA breaks. Michael et al. [28] find that TOP2β mediates DNA breaks through directly binding to androgen receptor at the position of androgen receptor downstream target gene promoters. We pre-retrieved and analyzed the corresponding ChIP-seq data through the Cistrome DB database (http://cistrome.org/). The results showed that TOP2β and CTCF were both found to bind to the *TFE3* gene and partner genes we were concerned about (PRCC, SFPQ, ASPSCR1, NONO, MED-15) [see Additional file 3]. Since TOP2β has been proved to mediate DNA breaks [29], we used TOP2β ChIP to demonstrate the binding of TOP2β at the translocation sites to prove that the *TFE3* gene could break at the translocation sites through TOP2β-mediated DNA breaks. And we used *TFE3* break-apart FISH to detect TFE3 breaks. The further ERα ChIP was also used to explore the potential effects of ERα on TOP2β at the translocation site. However, our study failed to find the direct bindings of ERα and TOP2β, we proposed that ERα and TOP2β could be co-recruited to translocation sites in the intron regions rather than only in the promoter regions. Based on the study that TOP2 cleaves DNA double strands to results in TOP2 cleavage complex (TOP2cc) through a transesterification reaction [30], we put forward hypothesis that, in a narrow spatial chromatin region, the binding of ERα with DNA sequence near the breaking site may have spatial-dependent harms on the biological functions of TOP2cc, making TOP2cc fall off from DNA strands, and finally resulting in DNA breaks. Thus, further research should be done to prove this hypothesis. And we found evidence for enrichment of CTCF at *TFE3* translocation sites, implying that *TFE3* translocation sites may located in anchors of chromatin loops [29]. This also explains why TFE3 translocation sites are more located in the intron 3–4 and intron 5–6, based on the DNA fragility caused by spatial chromosome folding [31, 32].

Studies confirm that TOP2-mediated DNA breaks acts primarily through transcription-dependent processes [30, 33], suggesting a higher risk of DNA breaks in highly transcribed genes. And the effect of estrogen on the occurrence of tumors has been widely concerned. Previous animal experiments have confirmed that estrogen can induce the occurrence of hamster kidney tumors [34]. Estrogen is an important reproductive endocrine hormone, which regulates the expression of thousands of genes by combining with ER. ERα expression predominant in uterus, pituitary, kidney, and adrenal gland and ERβ expression predominant in ovarian granulosa cells, prostate, bladder, and lung [35]. Among all genes regulated by estrogen, the number of genes expressed by the kidney ranks third, and even more than that of ovarian granulosa cells [36]. Therefore, we considered that E2 could upregulate *TFE3* expression by enhancing its transcriptional activity through NRF1 pathway, and consequently increase the risk of *TFE3* breaks. And finally, we found that E2 regulated *TFE3* transcription via NRF-1. As the publication work of the detailed research on the regulation of *TFE3* transcription by NRF1 is in progress, only a brief verification was provided in this study. On the other hand, when reviewing the transcript levels of fusion partners in Xp11.2 tRCC, we found that some of them were housekeeping genes with high transcriptional activity [37], such as *NONO* and *CLTC*. This caused that these fusion partners are more accessible to face DNA breaks and chromosome translocations, which reflected that the seemingly random chromosome translocations might have an inevitable reason.

Besides, based on the specificity of the *TFE3* gene, studies have confirmed that TFE3 can be activated during DNA damage, and play an important role by directly regulating p53, causing cell cycle arrest or apoptosis [38]. Which means the changes in TFE3 protein function by the translocation at Xp11.2 can further aggravate the consequences of DNA damage, and thus played an important role in this process of tumorigenesis.

In a sense, this study offers a method for predicting translocation sites in Xp11.2 tRCC. Our data indicated that ERα and TOP2β were co-recruited to translocation sites not only in *TFE3* gene, but also in fusion partner genes. Conversely, we can explore new fusion partners or translocation sequences by analyzing the intersection between ERα-bound sequence and TOP2β-bound sequence. However, the generation of tRCC involves not only the occurrence of DNA breaks, but also obstacles to the double-strand repair mechanism. Considering that this was a multi-factor participation process, other factors such as gene mutation, sudden changes in hormone levels were also play an important role. The pathogenesis of tRCC cannot not be predicted by ERα and TOP2β alone. Through further studies of potential translocation sequence in fusion partners, the rules governing fusion partner choice in Xp11.2 tRCC may be delineated.

## Conclusions

This study indicates that E2 amplifies TOP2β-mediated *TFE3* breaks by ERα-dependent pathway, and E2 upregulates *TFE3* by NRF1 to increase the risk of *TFE3* breaks. Which suggests that E2 is an important pathogenic factor for Xp11.2 tRCC pathogenesis.

## Supplementary Information

**Additional file 1**. Sequences of probes, primers, siRNAs and shRNAs used, and translocation sites metioned in this study.

**Additional file 2**. Results of putative transcription factors that can bind to the promoter region of TFE3.

**Additional file 3**. ChIP-seq data analysis from Cistrome DB database.

**Additional file 4**. **Figure S1**. Supplementary figures. (**A**) Detection of Cell Counting Kit-8 (CCK8) cell viability in HK-2 cells under 0.1% DMSO (solvent-only control), 50 μM, 75 μM, 100 μM, 125 μM or 150 μM etoposide treatment for 1 h. (**B**) Western blot detection of TOP2α and TOP2β in HK2 cells (three biological replicates). (**C**) Knockdown efficiency of siTOP2A and siTOP2B detected by qPCR. (**D**) Representative image of micronuclei (pointed by the yellow arrow). (**E**) Western blot of total protein of HK-2 cells detected with anti-ERα antibodies (Three biological replicates). (**F**) Immunofluorescence assay of ERα in HK-2 cells. (**G**) Knockdown efficiency of shESR1 and shESR2 detected by qPCR. (**H**) Knockdown efficiency of shNRF1 detected by qPCR. Error bars indicate 95% confidence intervals (** *p* < 0.01, *** *p* < 0.001).

## Data Availability

Supporting data were available to all researchers.

## Abbreviations

tRCC: Translocation renal cell carcinoma
TFE3: Transcription factor E3
TOP2: Topoisomerase II
E2: 17β-Estradiol
ER: Estrogen receptor
BPE: Bovine pituitary extract
EGF: Epidermal growth factor
FISH: Fluorescence in-situ hybridization
ChIP: Chromatin immunoprecipitation
Co-IP: Co-immunoprecipitation
γ-H2AX: Phosphorylated H2AX
MN: Micronuclei
NRF1: Nuclear respiratory factor 1
TOP2cc: TOP2 cleavage complex
