# Supplementary material for: Estradiol increases risk of topoisomerase IIβ-mediated DNA strand breaks to initiate Xp11.2 translocation renal cell carcinoma
Source: Cell Commun Signal. 2021 Nov 16;19:114. doi: 10.1186/s12964-021-00790-3 (PMC8594210; doi:10.1186/s12964-021-00790-3)
Supplement: Supplementary file 2 — Additional file 1. Sequences of probes, primers, siRNAs and shRNAs used, and translocation sites metioned in this study. [file 12964_2021_790_MOESM2_ESM.docx]

1. Probe sequences used in target region sequencing
2. Probe 1: CTGCATGTGTTTCTTGCCTTCCTGTGCTCAGAGGCCCTGATCACTCCACTCTTTTCCAGAGACCGAGGCAAAGGCCCTTTTGAAGGAACGGCAGAAGAAAGACAATCACAACCTAAGTAA
3. Probe 2: AAGGCACTTTTTAAAAAAGGCATTTCCCCTGCTTTTGGCTACCAAACCCTACCTTAGTATCTGCATGTGTTTCTTGCCTTCCTGTGCTCAGAGGCCCTGATCACTCCACTCTTTTCCAGA
4. Probe 3: AGTTCCTCTCGTCTTGGATTTAACAACCAGCCTATCACCATGGCTTAAAATAGTAAAAAGAAGGCACTTTTTAAAAAAGGCATTTCCCCTGCTTTTGGCTACCAAACCCTACCTTAGTAT
5. Probe 4: AATGGAGGCTGTTTGTCCCCTTTCCCCCTATGGCTTGTGGTCTTGCTTTCACCCCACTTCAGTTCCTCTCGTCTTGGATTTAACAACCAGCCTATCACCATGGCTTAAAATAGTAAAAAG
6. Probe 5: CTGTGCCTACCAGGTCTAGAGATGGGCCTGATTCTGAGGGGAATGGTTTGGACATATGGAAATGGAGGCTGTTTGTCCCCTTTCCCCCTATGGCTTGTGGTCTTGCTTTCACCCCACTTC
7. Probe 6: GATCTCTGGTAGGTGATAGGGTCTTGGCCCCATGGCCCTACACTTTACCCCAGGTGAGCCCTGTGCCTACCAGGTCTAGAGATGGGCCTGATTCTGAGGGGAATGGTTTGGACATATGGA
8. Probe 7: GGAGGAGCCAAGCCTGGGTTTGTTCCCAACTGGTGGTTCTCTGCCCTCTCTGCCCCCAGCTGCCTGTGTCAGGGAATCTGCTTGATGTGTACAGTAGTCAAGGCGTGGCCACACCAGCCA
9. Probe 8: CCTTGCATTAGAGGGTGCCCCATCTTTGGGATATGTGGAAGCTCAGGAACCTGGTTCTGAGGAGGAGCCAAGCCTGGGTTTGTTCCCAACTGGTGGTTCTCTGCCCTCTCTGCCCCCAGC
10. Probe 9: GGTCGCCATGTGAATTTGCCTTGAAGGAGAAGCAAGGGTTCTGGTGCTGAGTCAGCCAGTCCTTGCATTAGAGGGTGCCCCATCTTTGGGATATGTGGAAGCTCAGGAACCTGGTTCTGA
11. Probe 10: GGCAGGCTCCAGTCTCCCAGCGAAGCAAGGTAGGTCCAGTTTGCTCTGTCCTCCCTGTTTGGTCGCCATGTGAATTTGCCTTGAAGGAGAAGCAAGGGTTCTGGTGCTGAGTCAGCCAGT
12. Probe 11: AAAGGGGCTTTAGAGGTCACTGAGGGATATGAGGGACTGGAGGAAGTGATAAGCAGGCCAGGCAGGCTCCAGTCTCCCAGCGAAGCAAGGTAGGTCCAGTTTGCTCTGTCCTCCCTGTTT
13. Probe 12: AAAGACCTGAGAAGGGTGGTTTTCCAGCATTTCTTCTCAGAGCCAGCCCCGGGGTTTGGGAAAGGGGCTTTAGAGGTCACTGAGGGATATGAGGGACTGGAGGAAGTGATAAGCAGGCCA
14. Probe 13: CTGGATTGGAATGTTGTGCTGGGAGGAGCTAACGGACTGGAATGTCCCATCAGAGCCGACAAAGACCTGAGAAGGGTGGTTTTCCAGCATTTCTTCTCAGAGCCAGCCCCGGGGTTTGGG
15. Probe 14: AATGTTGTCATGACAATTTACCAAGGTCGGAGAAGATTGGGTTGGAGTGTTCCAAGAATGCTGGATTGGAATGTTGTGCTGGGAGGAGCTAACGGACTGGAATGTCCCATCAGAGCCGAC
16. Probe 15: GATCAGAGTGAAAGACTGGAGATTAGGTTGGAATTCTCCAGAAGGCCAGATTTTGGATAGAATGTTGTCATGACAATTTACCAAGGTCGGAGAAGATTGGGTTGGAGTGTTCCAAGAATG
17. Probe 16: GCAGGAGACCCTGATGTGGAAGATGGTACAACTTGATCAGAGTGGAAGATGGTACAACTTGATCAGAGTGAAAGACTGGAGATTAGGTTGGAATTCTCCAGAAGGCCAGATTTTGGATAG
18. Probe 17: ATCTCCTGAGAGAACAGAATGTGCAGGAAGATACTGGAGTCAGGACCTTGGTCCACAGCAGCAGGAGACCCTGATGTGGAAGATGGTACAACTTGATCAGAGTGGAAGATGGTACAACTT
19. Probe 18: GAGGAAAAAAAAAAAAACTAGGCTGACATAAAGCAGTGCTAATTGAATAGTTGGATAAGAATCTCCTGAGAGAACAGAATGTGCAGGAAGATACTGGAGTCAGGACCTTGGTCCACAGCA
20. Probe 19: CCCTGGGTATACTTAAGATTGACGTGCTCCTCTGGAGTATATCCAGGAAAGTGTGAAGGAGAGGAAAAAAAAAAAAACTAGGCTGACATAAAGCAGTGCTAATTGAATAGTTGGATAAGA
21. Probe 20: GGGATTACAGGCGTGAGCCACTGCACCTGGCCCTGGGTATACTTAAGATTGACGTGCTCCTCTGGAGTATATCCAGGAAAGTGTGAAGGAGAGGAAAAAAAAAAAAACTAGGCTGACATA
22. Probe 21: ACTAATTTATCAGAACTTTTTATTGATTTGGACACAACTAGAGAATGGGACCTTATATCCACCAGACTGGAAGTGGGTTCAAGAGATTAAAGCTCAGCCAGGCACGGTGGCTCACACCTG
23. Probe 22: TGGCTGTAGTTTTGCTTTTTTCCAACTTATCAATAGGATGGAAGCATGTATAAAAATGGGACTAATTTATCAGAACTTTTTATTGATTTGGACACAACTAGAGAATGGGACCTTATATCC
24. Probe 23: CTGGGATTACAGACATAGCCACCATGCCTGGTCTCATAACTTGCTTTAGAGCATACCAAGTGGCTGTAGTTTTGCTTTTTTCCAACTTATCAATAGGATGGAAGCATGTATAAAAATGGG
25. Probe 24: GAACTGTGCCAGGAGGCAGAGTCCTTCTAGTCAGTAGATAAAACTTTCTAGAGCAAGGGATATACCATTGTAACATTCTGGTTTTGAACACTCCAAGAGTCTCATAACTTGCTTTATTAT
26. Probe 25: CAAACTTGCTGTGGGTTATAACAAGTAGTTGGATCTCGTAGTGATTGGAATATACCAAGTGAACTGTGCCAGGAGGCAGAGTCCTTCTAGTCAGTAGATAAAACTTTCTAGAGCAAGGGA
27. Probe 26: AAGTGCTGGAATTACAGGTGTGAGCCACCACACCTGGCCTTGAAGCATAACAAAAGTTTTCAAACTTGCTGTGGGTTATAACAAGTAGTTGGATCTCGTAGTGATTGGAATATACCAAGT
28. Probe 27: CCGAGATGAGCATCTTATAGCAACTTGAATGTACCAAGGGATTCAGGTCATCGGGACATATACACCAGACCATGTAATGATTGGAGCATTTTGTTTTATTTTATTTTTACCTTTTTTTTC
29. Probe 28: GGGATTACAGGCGTGAGCCACCACACCCGGCCGAGATGAGCATCTTATAGCAACTTGAATGTACCAAGGGATTCAGGTCATCGGGACATATACACCAGACCATGTAATGATTGGAGCATT
30. Probe 29: TTGGACTGTGTTGAGAGGTGGACCATGTAGACTGTGAGAGATACAGGTGGGGAGTTCACTGAGACAGTAGGGCATATCAAAGTGCTTGGGCTTTGCTGTGAGAAGTACCAAGAGATGATA
31. Probe 30: AGATAGAGAGGGAGGCCTTATGACGAGAACAGGGTATGTCAACAATGCCTTTGGACCTTGTTGGACTGTGTTGAGAGGTGGACCATGTAGACTGTGAGAGATACAGGTGGGGAGTTCACT
32. Probe 31: TCACCAAGACCATACTCAGTCTGGCTGTGTGTATAGGAGACCCTTGCCTTGATTGTGCTAAGATAGAGAGGGAGGCCTTATGACGAGAACAGGGTATGTCAACAATGCCTTTGGACCTTG
33. Probe 32: TCCCCAGCACGGTGAGGCCCTGAGATGGGAGGTTGGTCTGAAAATTAGGGCATTTCTGTATCACCAAGACCATACTCAGTCTGGCTGTGTGTATAGGAGACCCTTGCCTTGATTGTGCTA
34. Probe 33: AGTCCAGTTACAATGATGAAATGCTCAGCTATCTGCCCGGAGGCACCACAGGACTGCAGCTCCCCAGCACGGTGAGGCCCTGAGATGGGAGGTTGGTCTGAAAATTAGGGCATTTCTGTA
35. Probe 34: TCCCCTCCCAAATTCTTCTAAACCTGTATAATATTTACTTCTTCCCCTAGATTGATGATGTCATTGATGAGATCATCAGCCTGGAGTCCAGTTACAATGATGAAATGCTCAGCTATCTGC
36. Probe 35: CACCATCGGGTCCAGCTCAGAGAAGGAGGTAAGAGGCTACAGCCAAACCTCCTCCCACATTCCCCTCCCAAATTCTTCTAAACCTGTATAATATTTACTTCTTCCCCTAGATTGATGATG
37. Translocation sites of UOK109, UOK120 cells and 10 Xp11.2 translocation RCC patients.

|  | Fusion partner | | TFE3 |
| --- | --- | --- | --- |
|  | Name | Translocation site | Translocation site |
| UOK109 | NONO | chrX: 70517925 | chrX: 48893631 |
| UOK120 | PRCC | chr1: 156746784 | chrX: 48895985 |
| Patient 1 | NONO | chrX: 70517956 | chrX: 48894282 |
| Patient 2 | PRCC | chr1: 156762901 | chrX: 48895666 |
| Patient 3 | SFPQ | chr1: 35648821 | chrX: 48892309 |
| Patient 4 | SFPQ | chr1: 35644377 | chrX: 48893346 |
| Patient 5 | SFPQ | chr1: 35652211 | chrX: 48895530 |
| Patient 6 | SFPQ | chr1: 35646901 | chrX: 48895554 |
| Patient 7 | ASPSCR1 | chr17: 79960167 | chrX: 48892023 |
| Patient 8 | ASPSCR1 | chr17: 79962182 | chrX: 48895784 |
| Patient 9 | ASPSCR1 | chr17: 79960905 | chrX: 48891946 |
| Patient 10 | MED15 | chr22: 20927905 | chrX: 48893163 |

Chromosome positions are marked according to ENSG database.

1. The primers and primer sequences used were as follows.
   1. *NONO*
      1. For translocation site of **UOK109**:
         1. Forward: CGGCAGCAAGAAGAAATGATG;
         2. Reverse: GGGAAAATTGGTCATAACCGG;
      2. For translocation site of **Patient 1**:
         1. Forward: ATATGCAACCTTGGCTAGTCCTC;
         2. Reverse: CAAGGACCACACACTAAGGGA;
   2. *PRCC*
      1. For translocation site of **UOK120**:
         1. Forward: TTGTTCTGTGTGGTCTGCCA;
         2. Reverse: AGACTTAAGCACCTGGCACC;
      2. For translocation site of **Patient 2**:
         1. Forward: CCAGAAAGGTGTCCATCCCT;
         2. Reverse: TTCAGTGAGACCCAGTCCTGA;
   3. *SFPQ*
      1. For translocation site of **Patient 3**:
         1. Forward: GCTACTTCTGTTGGGCAAAATCT;
         2. Reverse: TTCCAAGCCTTTATGGTGGGAG;
      2. For translocation site of **Patient 4**:
         1. Forward: TGTCAGCTAGTGCAGTTCTCA;
         2. Reverse: TGTCCTATCTTAAGTCCAAGCCC;
      3. For translocation site of **Patient 5**:
         1. Forward: CGGTTGCCAACAAGGAGTCA;
         2. Reverse: CCAGCTTAAAGAATGAGTTTGCCA;
      4. For translocation site of **Patient 6**:
         1. Forward: AGAAGTACTACAAACCCCACCAC;
         2. Reverse: CAGAAAACATTCCGCCACAACT;
   4. *ASPSCR1*
      1. For translocation site of **Patient 8**:
         1. Forward: CCCTCAGAGACGCTGGAAAC;
         2. Reverse: TCACAGCAGACAGTGCTGG;
   5. *MED15*
      1. For translocation site of **Patient 10**:
         1. Forward: CTTAAGCTTTGCATTCCTTGCG;
         2. Reverse: AGCAACAGATGAACCGACCG;
   6. *TFE3*
      1. For translocation site of **UOK109**:
         1. Forward: TAACTGGGACCATAGGCTTGTG;
         2. Reverse: ACAGCCACTTGGTATGCTCTAAA;
      2. For translocation site of **UOK120**:
         1. Forward: ACGAGGGATCCTGTCAGTCAT;
         2. Reverse: TAGACTCACGCAGACAAGCTG;
      3. For translocation site of **Patient 1**:
         1. Forward: AGTTTTCAAACTTGCTGTGGGT;
         2. Reverse: GCAAGTTATGAGACTCTTGGAGTG;
      4. For translocation site of **Patient 2**:
         1. Forward: AGTGCGCCCAACAGCC;
         2. Reverse: TTTGGGAGGGGAATGTGGGA;
      5. For translocation site of **Patient 3**:
         1. Forward: TTGACGTGCTCCTCTGGAGT;
         2. Reverse: TCTTCCACATCAGGGTCTCCT;
      6. For translocation site of **Patient 5** and **Patient 6**:
         1. Forward: AGGAGACCCTTGCCTTGATTG;
         2. Reverse: ACTTCTCACAGCAAAGCCCA;
      7. For translocation site of **Patient 7**:
         1. Forward: CTCCCAGCGAAGCAAGGTAG;
         2. Reverse: CCAGGTTCCTGAGCTTCCAC;
      8. For translocation site of **Patient 8**:
         1. Forward: CTTATTTCACAGGTGCAGACCCA;
         2. Reverse: CGAGTGTGGTGGACAGGTACT;
      9. For translocation site of **Patient 9**:
         1. Forward: TCCTTGCATTAGAGGGTGCC;
         2. Reverse: TGACAGTGATGGCTGGTGTG;
   7. *ACTB*:
      1. Forward: CTCGCCTTTGCCGATCC;
      2. Reverse: TTCTCCATGTCGTCCCAGTT;
   8. *TOP2A*:
      1. Forward: AAGTGTCACCATTGCAGCCT;
      2. Reverse: ACCCACATTTGCTGGGTCAC;
   9. *TOP2B*:
      1. Forward: TGCCGTGCATTAGCACAGAT;
      2. Reverse: AGGTGTGTGGGAGCTTTTACT;
   10. *ESR1*:
       1. Forward: TTCCCTACCGCCTCCACTC;
       2. Reverse: CACTTTTCCAAACAAGACATACCA;
   11. *ESR2*:
       1. Forward: TTCAAAGAGGGATGCTCACTTC;
       2. Reverse: CCTTCACACGACCAGACTCC;
   12. *TFE3*:
       1. Forward: TGTTCGTGCTGTTGGAGGAG;
       2. Reverse: TCCTGGAGCCCCCTTGAG;
   13. *NRF1*:
       1. Forward: GTGGTATGCTGACATTTAAACAGG;
       2. Reverse: CAGCTGCTGTGGAGTTGAGTA;
2. The siRNA and siRNA sequences used were as follows.
   1. NC: UUCUCCGAACGUGUCACGUTT;
   2. siTop2A: GGAGAAGAUUAUACAUGUAUC;
   3. siTop2B: GGUGUAUGAUGAAGAUGUAGG
3. The shRNA and shRNA sequences used were as follows.
   1. NC: GCCTAAGGTTAAGTCGCCCTCGCTCGAGCGAGGGCGACTTAACCTTAGG
   2. shESR1: CTACAGGCCAAATTCAGATAACTCGAGTTATCTGAATTTGGCCTGTAG;
   3. shESR2: GCGAGTAACAAGGGCATGGAACTCGAGTTCCATGCCCTTGTTACTCGC;
   4. shNRF1: CACATTGGCTGATGCTTCATTCTCGAGTGAAGCATCAGCCAATGTGTT;
