# Supplementary material for: Estradiol increases risk of topoisomerase IIβ-mediated DNA strand breaks to initiate Xp11.2 translocation renal cell carcinoma
Source: Cell Commun Signal. 2021 Nov 16;19:114. doi: 10.1186/s12964-021-00790-3 (PMC8594210; doi:10.1186/s12964-021-00790-3)
Supplement: Supplementary file 4 — Additional file 3. ChIP-seq data analysis from Cistrome DB database. [file 12964_2021_790_MOESM4_ESM.docx]

ChIP-seq data analysis from Cistrome DB database (<http://cistrome.org/>).

Since there was no TOP2β ChIP-seq public data of human kidney-derived cells or tissues in the database, we included three kinds of mouse TOP2β ChIP-seq data, as well as the only two human TOP2β ChIP-seq data. And other three CTCF ChIP-seq public data in the human kidney-derived cell line HEK293 were also included.

TOP2β ChIP-seq in Mus musculus


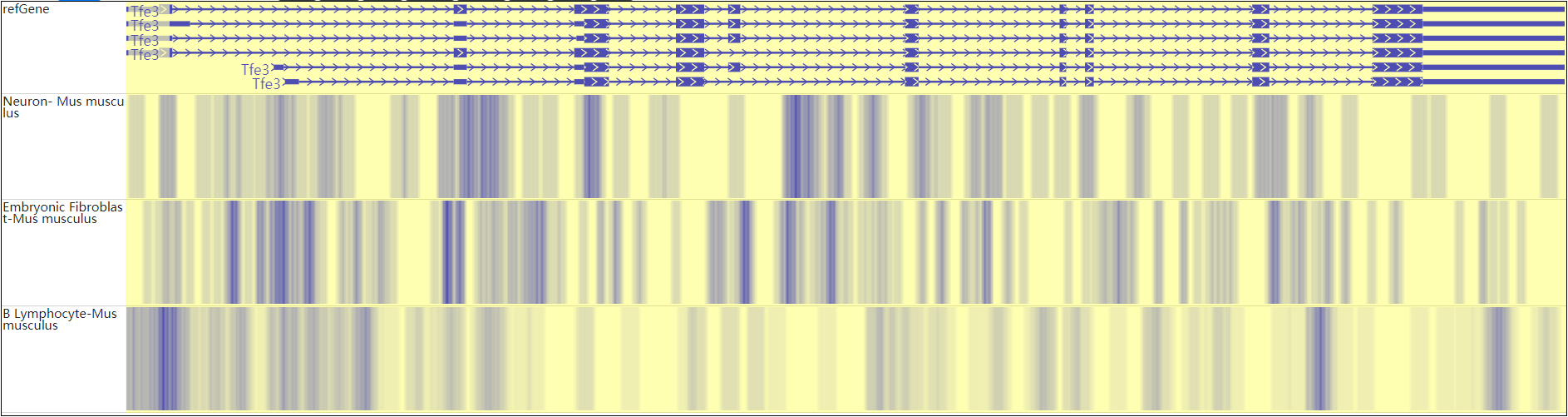
TFE3


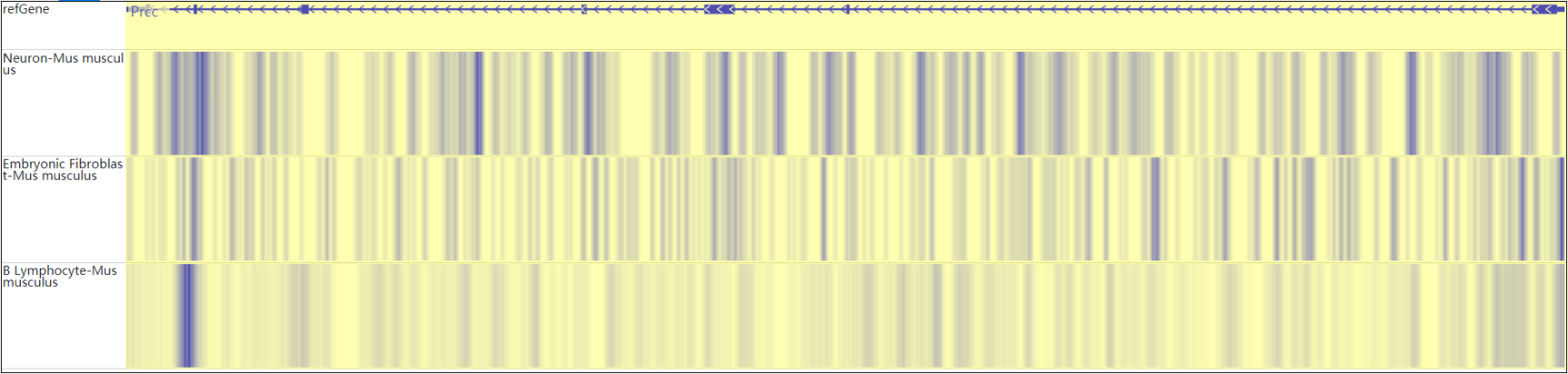
PRCC


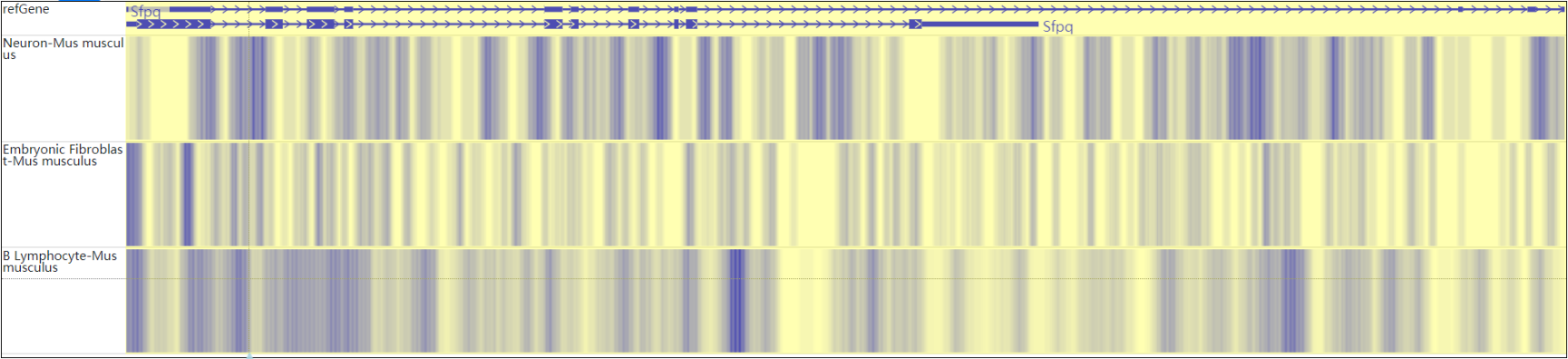
SFPQ


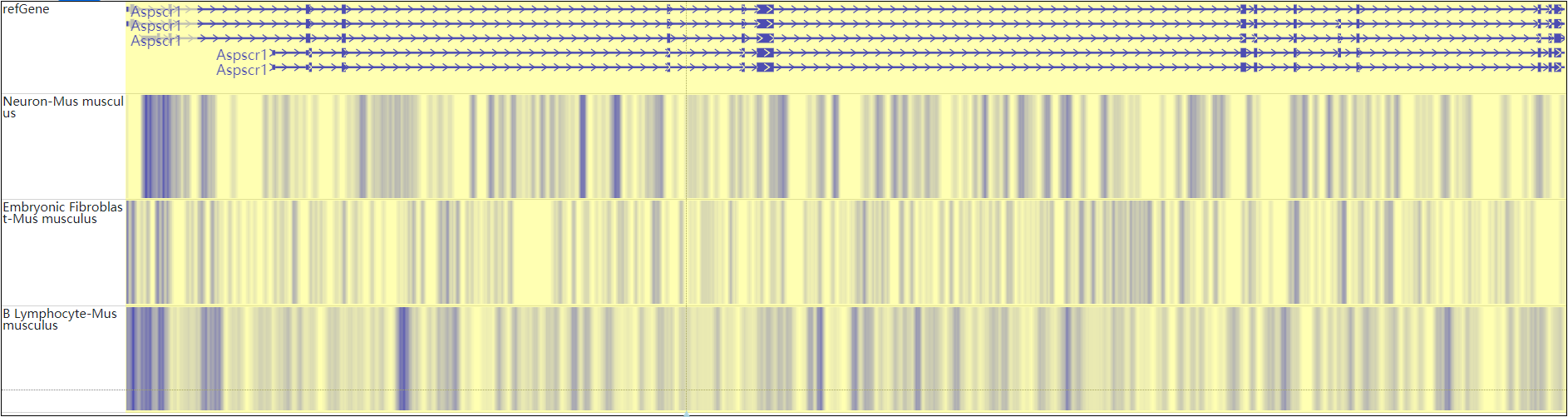
ASPSCR1


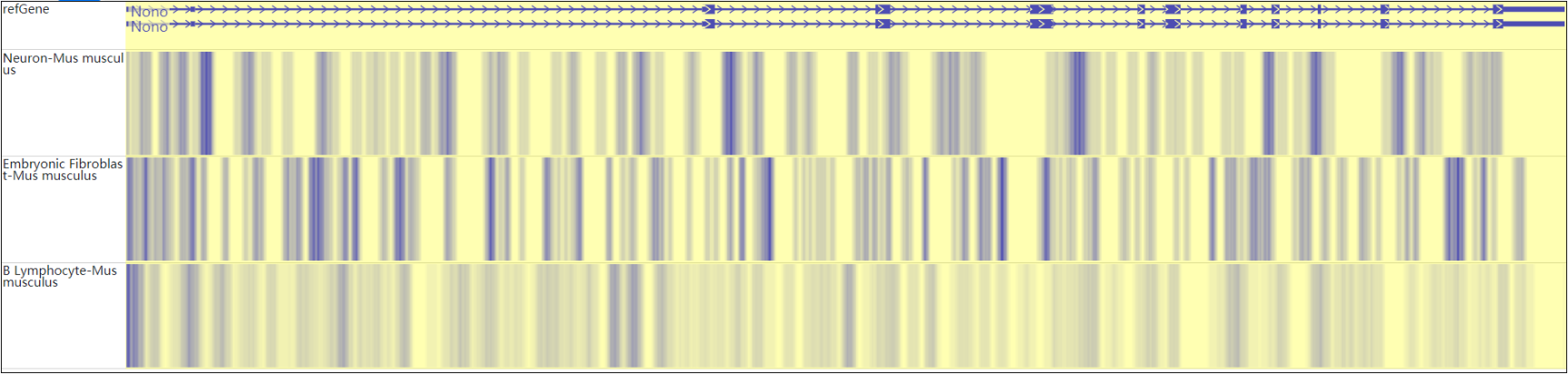
NONO


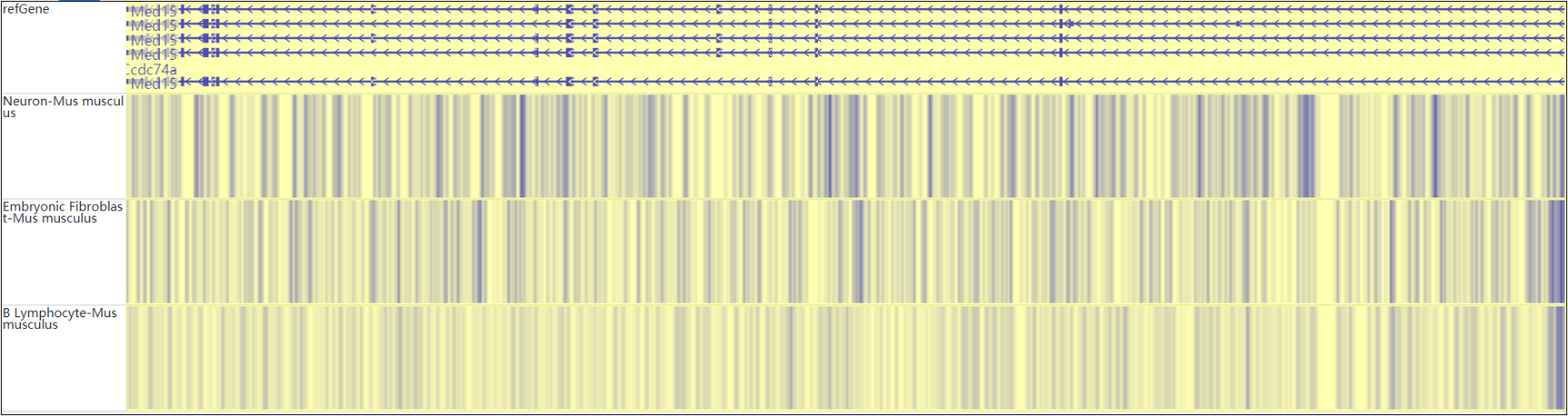
MED15

TOP2β ChIP-seq in Homo sapiens


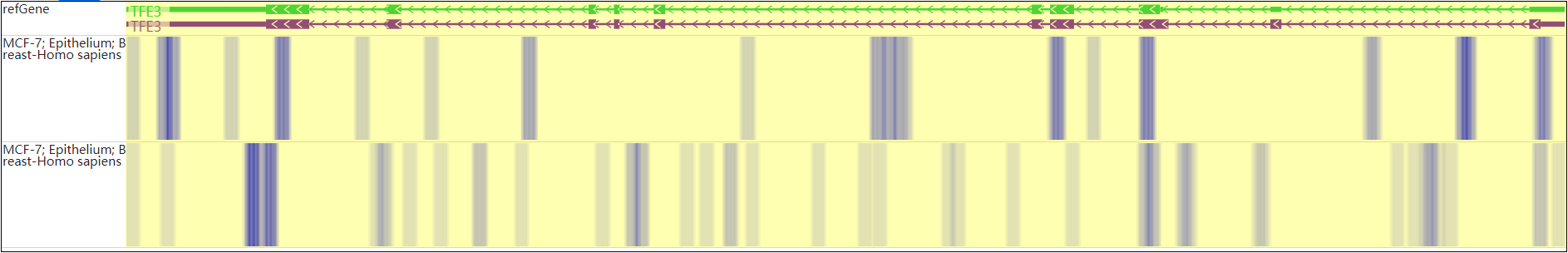
TFE3


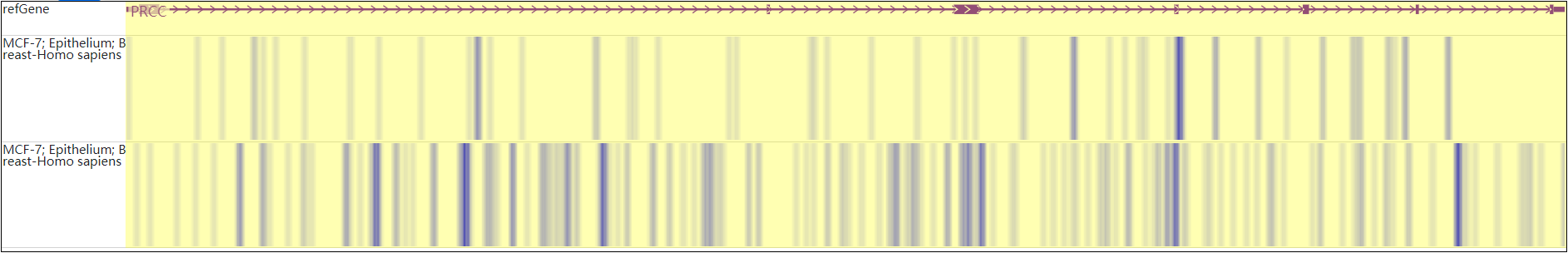
PRCC


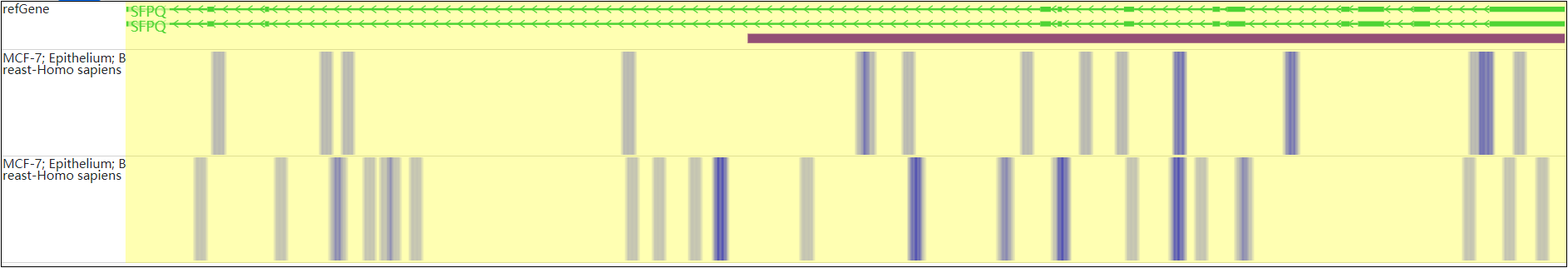
SFPQ


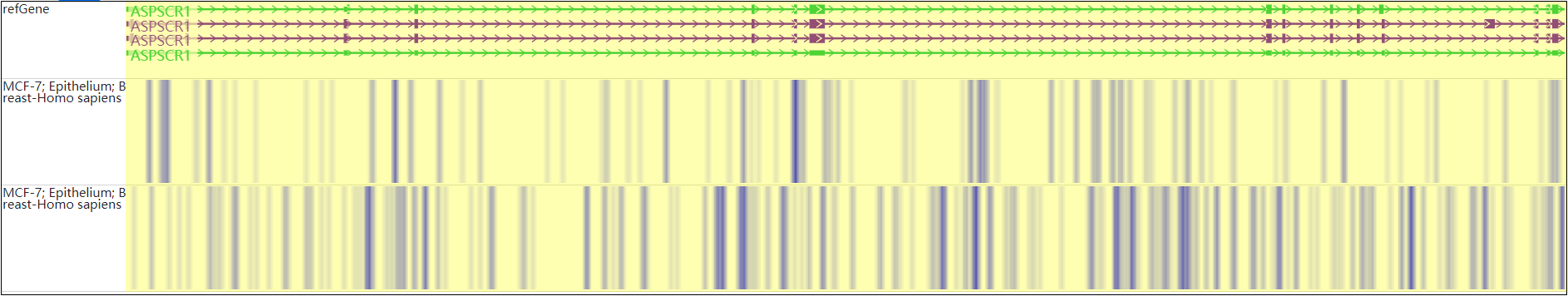
ASPSCR1


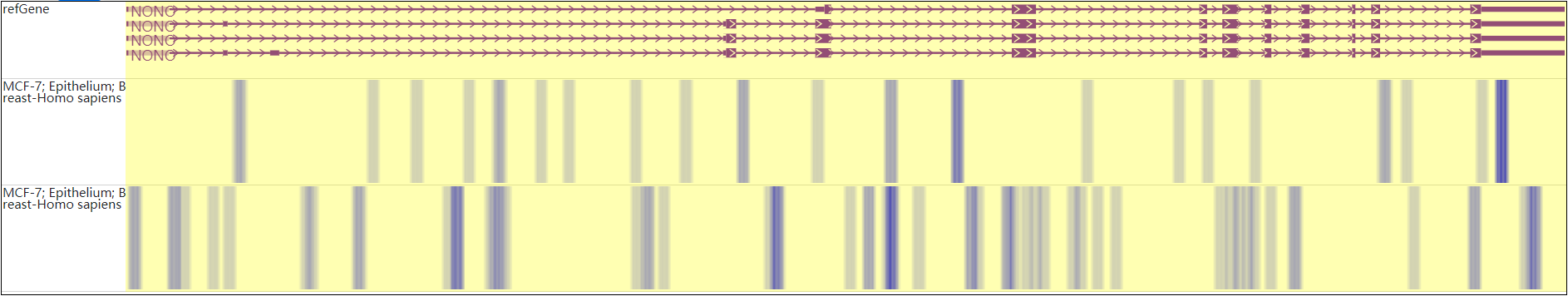
NONO


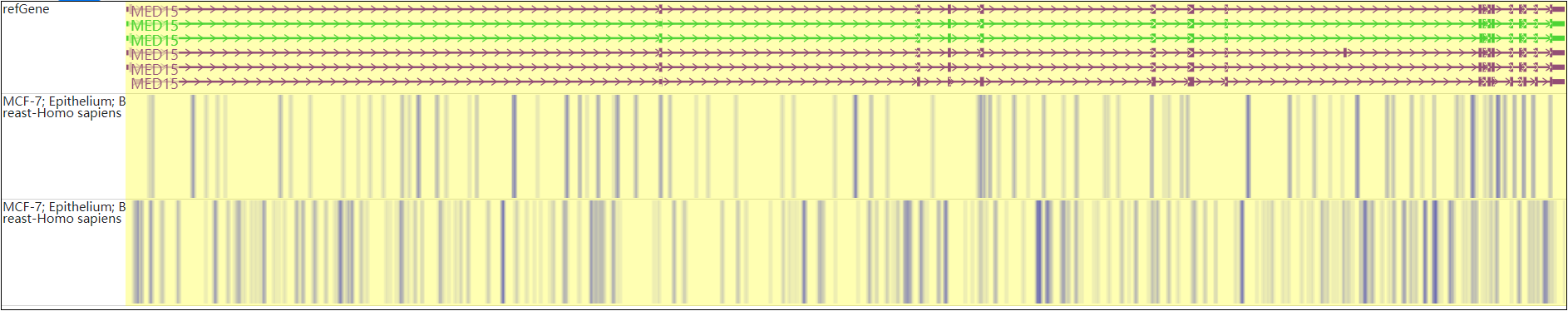
MED15

CTCF ChIP-seq in Homo sapiens HEK293


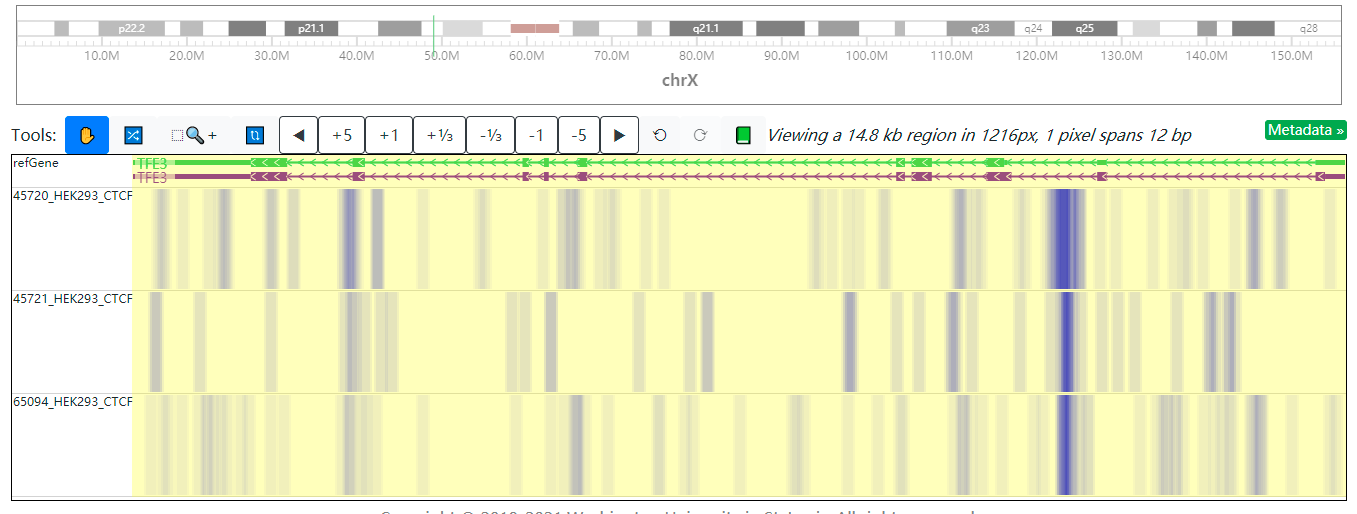
TFE3


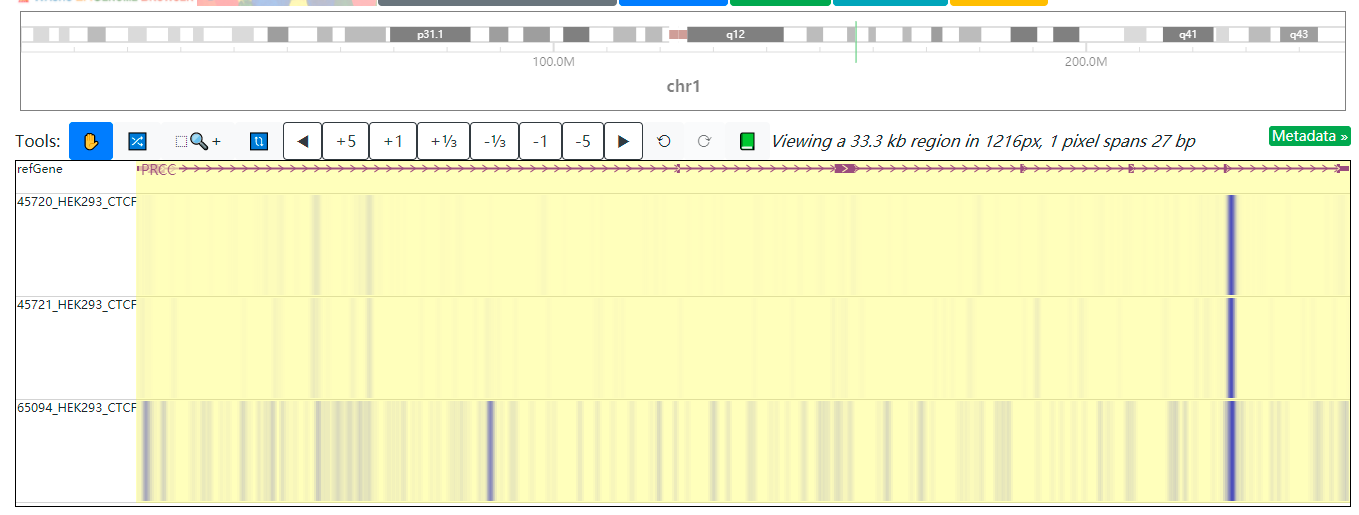
PRCC

SFPQ


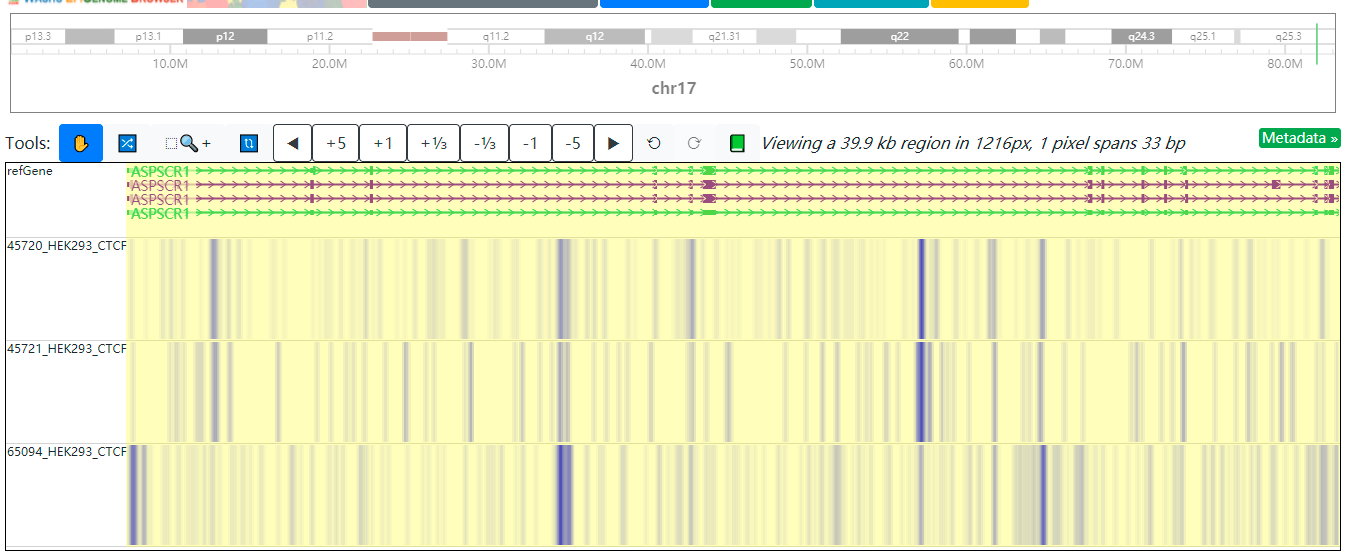

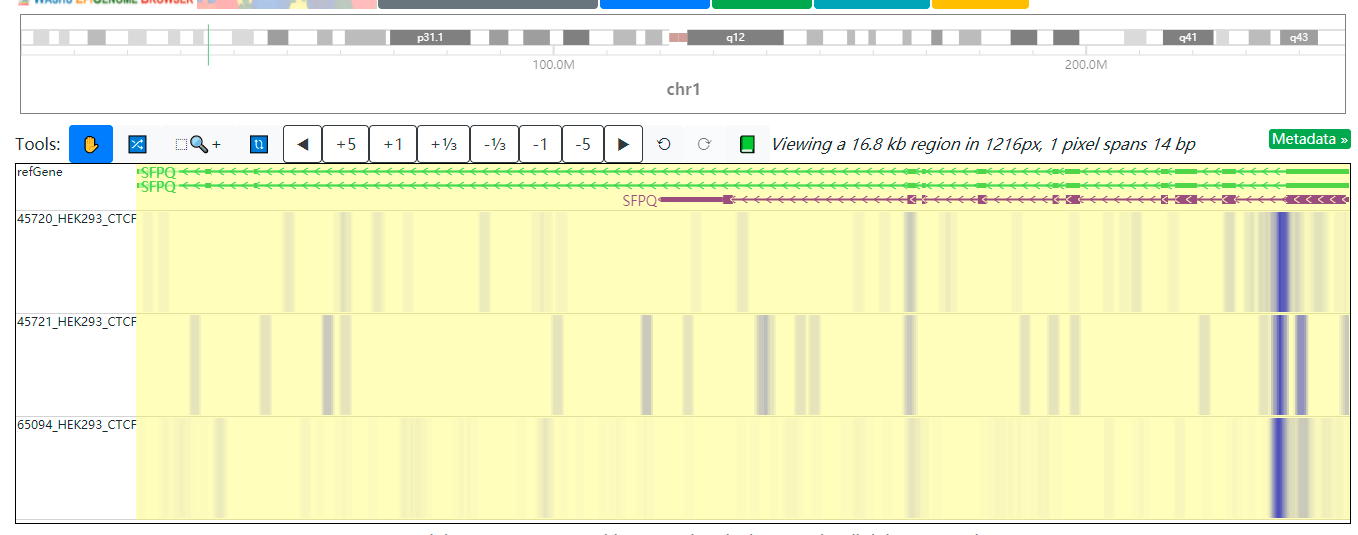
ASPSCR1


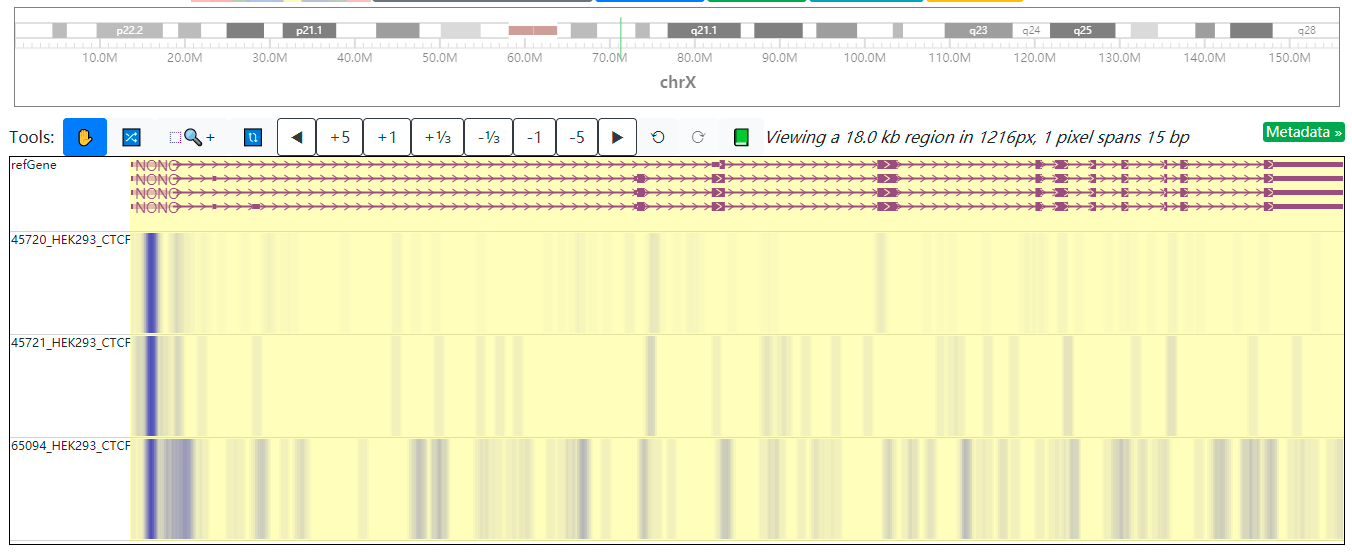
NONO


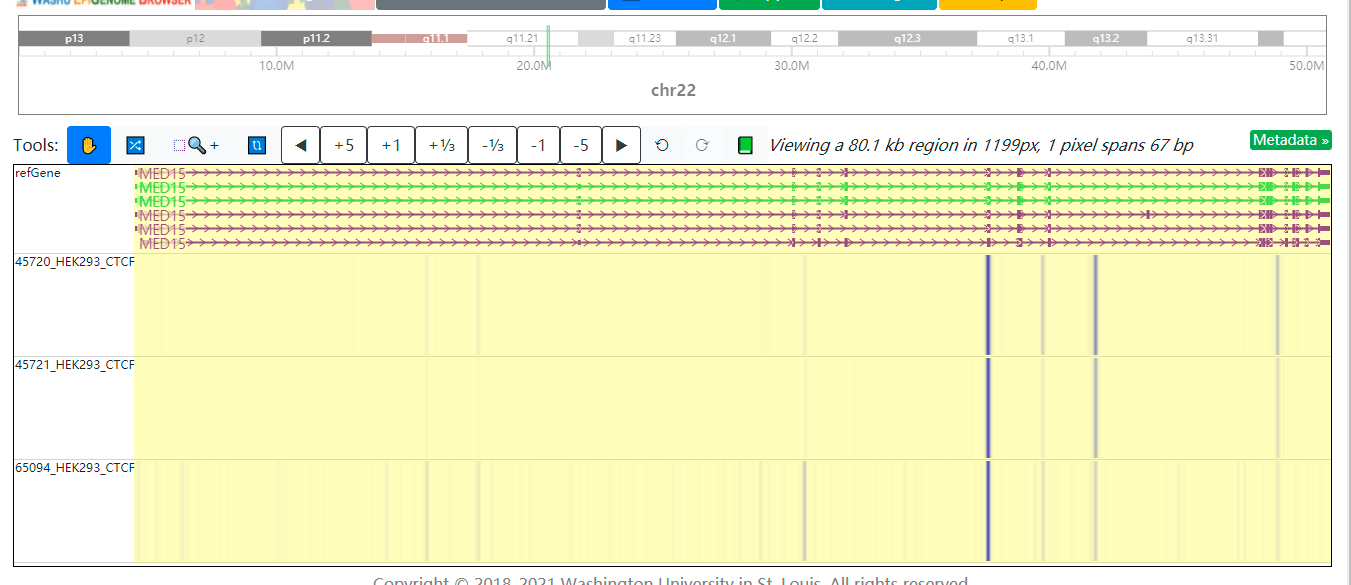
MED15
