# Supplementary figures and images for: Estradiol increases risk of topoisomerase IIβ-mediated DNA strand breaks to initiate Xp11.2 translocation renal cell carcinoma
Source: Cell Commun Signal. 2021 Nov 16;19:114. doi: 10.1186/s12964-021-00790-3 (PMC8594210; doi:10.1186/s12964-021-00790-3)

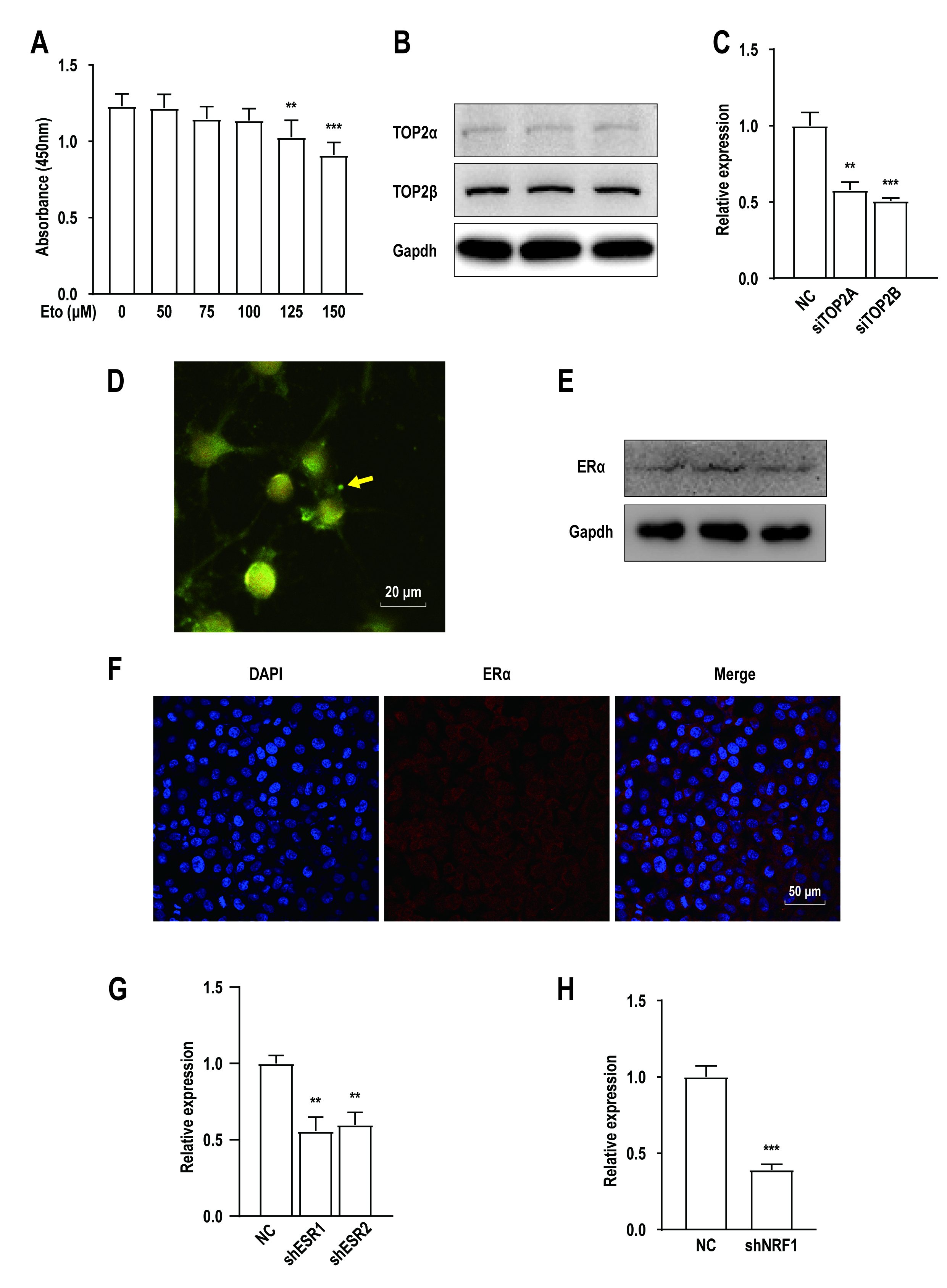

Supplement: Supplementary file 5 — Additional file 4. Figure S1. Supplementary figures. (A) Detection of Cell Counting Kit-8 (CCK8) cell viability in HK-2 cells under 0.1% DMSO (solvent-only control), 50 μM, 75 μM, 100 μM, 125 μM or 150 μM etoposide treatment for 1 h. (B) Western blot detection of TOP2α and TOP2β in HK2 cells (three biological replicates). (C) Knockdown efficiency of siTOP2A and siTOP2B detected by qPCR. (D) Representative image of micronuclei (pointed by the yellow arrow). (E) Western blot of total protein of HK-2 cells detected with anti-ERα antibodies (Three biological replicates). (F) Immunofluorescence assay of ERα in HK-2 cells. (G) Knockdown efficiency of shESR1 and shESR2 detected by qPCR. (H) Knockdown efficiency of shNRF1 detected by qPCR. Error bars indicate 95% confidence intervals (** p < 0.01, *** p < 0.001). [file 12964_2021_790_MOESM5_ESM.tif]
